# Extreme atherogenic index of plasma (log[TG_max/HDL_min]) and prognosis in sepsis-associated acute kidney injury: A MIMIC-IV retrospective study

**DOI:** 10.1097/MD.0000000000049718

**Published:** 2026-07-10

**Authors:** Siyao Zeng, Hanxin Liu, Zhen Quan, Zhipeng Yao, Yu Zhang, Yue Li, Junbo Zheng, Hongliang Wang

**Affiliations:** a Harbin Medical University Graduate School, Harbin Medical University, Harbin, Heilongjiang, China; b Department of Critical Care Medicine, Second Affiliated Hospital of Harbin Medical University, Harbin, Heilongjiang, China; c Department of Pharmacology, College of Pharmacy, Harbin Medical University, Harbin, China.

**Keywords:** high-density lipoprotein cholesterol (HDL), length of stay (LOS), Log(TG_max/HDL_min), mortality, sepsis-associated acute kidney injury (SA-AKI), triglycerides (TG)

## Abstract

Sepsis-associated acute kidney injury (SA-AKI) substantially worsens clinical outcomes, yet reliable prognostic biomarkers remain limited. Dysregulation of lipid metabolism, particularly elevated triglycerides (TG) and decreased high-density lipoprotein cholesterol (HDL-C), may mirror the severity of critical illness. In this retrospective cohort study based on the Medical Information Mart for Intensive Care IV database, we investigated whether a logarithmic lipid ratio, log(TG_max/HDL_min), obtained during the first week of intensive care, predicts adverse outcomes among 1633 adults with SA-AKI. Higher log(TG_max/HDL_min) was independently associated with increased 1-year mortality and prolonged lengths of stay in both the intensive care unit and hospital after multivariable adjustment for age, sex, blood urea nitrogen, serum creatinine, and Sequential Organ Failure Assessment (SOFA) score. These associations were particularly pronounced in non-diabetic and insulin-naïve individuals. Restricted cubic spline analyses demonstrated a J-shaped relationship between the lipid ratio and mortality risk, and the model showed acceptable discrimination (area under the curve 0.816). These findings suggest that early elevation of log(TG_max/HDL_min) serves as a simple and accessible indicator of metabolic dysfunction and a potential risk marker associated with adverse outcomes in patients with SA-AKI. Further prospective studies are needed to confirm these associations and to explore potential clinical implications.

## 1. Introduction

Sepsis-associated acute kidney injury (SA-AKI) is the development of acute renal dysfunction in the context of sepsis.^[1]^ It is a common complication among critically ill septic patients, accounting for roughly 50% of acute kidney injury (AKI) cases in intensive care units (ICUs). Importantly, SA-AKI portends significantly worse outcomes than non-septic AKI.^[1]^ A recent multicenter study reported that 62.3% of ICU patients with sepsis developed SA-AKI, and those with severe SA-AKI (stage 2–3) had a markedly higher risk of in-hospital death.^[1]^ Overall, SA-AKI is associated with a several-fold increase in sepsis morbidity and mortality.^[2]^ Despite its prevalence and impact, effective targeted therapies for SA-AKI are lacking: management remains largely supportive, and prognosis is often poor.^[2]^

One proposed mechanism in SA-AKI pathogenesis is metabolic reprogramming, particularly involving altered lipid metabolism.^[3]^ In sepsis, renal tubular cells undergo a shift in energy production in response to stress. Under normal conditions, proximal tubules rely heavily on fatty acid oxidation (FAO) in mitochondria to meet their high adenosine triphosphate demands. During septic insult, however, FAO and oxidative phosphorylation are downregulated, forcing tubular cells to switch to less efficient glycolysis for ATP generation.^[3]^ While this switch (analogous to a “Warburg effect”) may initially help cells survive acute hypoxia or inflammatory stress, prolonged reliance on glycolysis can lead to energy deficiency and tubular cell injury, atrophy, or fibrosis.^[3]^ Additionally, impaired fatty acid metabolism causes lipid accumulation in the kidney; such lipotoxicity can trigger inflammatory responses and cell death, exacerbating AKI.^[4]^ Experimental evidence from our recent study further supports this concept: in a murine model of LPS-induced SA-AKI, we demonstrated that suppression of renal FAO and mitochondrial dysfunction are key drivers of tubular injury. Both pharmacological activation of lipid metabolism with fenofibrate and metabolic intervention with a ketogenic diet restored renal FAO, improved energy metabolism, and markedly attenuated renal injury. These findings collectively suggest that impaired lipid fuel utilization is a central feature of septic kidney injury and that restoration of FAO may represent a potential therapeutic strategy.^[5,6]^ Thus, severe dysregulation of lipid fuel utilization in the septic kidney is believed to be involved in SA-AKI development.

The ratio of triglycerides (TG) to high-density lipoprotein cholesterol (HDL) (TG/HDL), especially in log-transformed form (known as the atherogenic index of plasma [AIP]), is a recognized marker of systemic lipid dysregulation and insulin resistance.^[7]^ An elevated TG/HDL ratio has been associated with a variety of cardiometabolic conditions and adverse outcomes. For example, higher TG/HDL correlates with a greater incidence of metabolic syndrome, atherosclerotic cardiovascular diseases (CVD) (coronary, cerebrovascular, and peripheral arterial disease), and has been linked to worse outcomes such as increased mortality.^[8]^ Prior studies note that TG/HDL is often elevated in patients with insulin resistance or type 2 diabetes, and it predicts the development of diabetes and fatty liver disease more effectively than individual lipid measures.^[9]^ Moreover, elevated AIP (log[TG/HDL]) was recently shown to correlate with higher all-cause and cardiovascular mortality in populations with combined cardiovascular, kidney, and metabolic syndrome.^[7]^ In the context of acute critical illness, dyslipidemia also appears to play a role. Notably, the triglyceride–glucose (TyG) index, which incorporates TG as a marker of insulin resistance, was found to be strongly associated with SA-AKI occurrence and with longer ICU and hospital stays in septic patients.^[10]^ A higher TyG index has likewise shown a significant (nonlinear) association with greater short-term mortality in SA-AKI patients.^[11]^ On the other hand, low HDL cholesterol by itself is known to portend worse outcomes in sepsis; septic patients experience a 40 to 70% drop in HDL, and those with depressed HDL levels are at higher risk of AKI and death.^[12]^ These findings suggest that extreme derangements in lipid metabolism, high TG and low HDL during sepsis may reflect an atherogenic dyslipidemia that contributes to organ injury and poor prognosis. In light of this, we innovatively defined a new metric, log(TG_max/HDL_min), representing the logarithm of the highest TG value divided by the lowest HDL value observed during a patient’s ICU stay. This novel index captures the magnitude of extreme lipid disturbance in each SA-AKI patient. By integrating the maximum TG and minimum HDL levels within the first week of ICU admission, this index may reflect dynamic lipid disturbances in critically ill patients. In our study, we hypothesized that a higher log(TG_max/HDL_min) would be associated with worse outcomes. In this study, using data extracted from the Medical Information Mart for Intensive Care IV (MIMIC-IV) database, we aimed to investigate whether elevated log(TG_max/ HDL_min) is associated with adverse clinical outcomes in SA-AKI patients, including in-hospital, 28-day, 30-day, ICU, and 1-year mortality, as well as ICU and total hospital lengths of stay.

## 2. Materials and methods

### 2.1. Study design and data sources

This retrospective cohort study used data from the Medical Information Mart for Intensive Care IV (MIMIC-IV) database (version 3.1), which contains de-identified health records for ICU patients at Beth Israel Deaconess Medical Center from 2008 to 2022.^[13]^ Data use was approved with a waiver of informed consent.

Study population: We included patients who met the criteria for SA-AKI based on Sepsis-3 and Kidney Disease: Improving Global Outcomes (KDIGO) definitions. Specifically, sepsis was defined according to the Sepsis-3 consensus as life-threatening organ dysfunction due to infection, operationalized as an acute increase in Sequential Organ Failure Assessment (SOFA) score ≥2 points.^[14]^ AKI was defined by KDIGO criteria as either an acute rise in serum creatinine ≥0.3 mg/dL within 48 hours or ≥1.5 times baseline within 7 days, or a urine output <0.5 mL/kg/hour for ≥6 hours.^[15]^ We considered a case to be SA-AKI if the onset of AKI occurred within 7 days of sepsis onset, unifying the presence of both sepsis and AKI during the same admission.^[15]^ We identified all ICU patients in MIMIC-IV aged 18 years or older who fulfilled these Sepsis-3 and KDIGO criteria for SA-AKI. If patients had multiple ICU admissions, only the first ICU stay meeting the criteria was included to ensure independence of observations. Patients with missing data on TG or HDL needed for the exposure were excluded, as were those missing other key variables required for analysis.

### 2.2. Data collection

Data were extracted from the MIMIC-IV relational database using structured queries. We collected comprehensive information on patient characteristics, clinical measurements, and treatments during the ICU stay. Baseline demographic variables included age, sex, and race/ethnicity. We identified comorbid conditions from recorded diagnoses and past medical history, including chronic kidney disease (CKD), CVD, diabetes mellitus, congestive heart failure (CHF), chronic obstructive pulmonary disease (COPD), liver disease, cancer, and history of myocardial infarction (MI), among others. Acute illness severity was assessed using the SOFA score within the first 24 hours of ICU admission.

Laboratory measurements during the first week of ICU admission were obtained, including the maximum or minimum values of hemoglobin, white blood cell count, platelet count, absolute neutrophil count, absolute lymphocyte count, albumin, blood urea nitrogen (BUN), creatinine, calcium, potassium, alanine aminotransferase, aspartate aminotransferase (AST), prothrombin time, partial thromboplastin time, TG, total cholesterol, HDL, and low-density lipoprotein cholesterol. TG and HDL measurements were extracted from routine laboratory tests performed during the first ICU week. TG_max and HDL_min were defined as the highest TG value and lowest HDL value recorded within the first 7 days after ICU admission. This time window was selected to capture metabolic disturbances occurring during the acute phase of SA-AKI. The natural logarithm was used in the primary analysis. All lipid values used to derive TG_max and HDL_min were obtained during the ICU stay prior to outcome ascertainment. We also recorded interventions and treatments received in the ICU, such as use of invasive mechanical ventilation (MV), vasopressors support, renal replacement therapy (RRT), and medications including nephrotoxic drugs (e.g., certain antibiotics or contrast agents), insulin therapy, systemic glucocorticoids, inotropes (e.g., dopamine, dobutamine), diuretics, and β-adrenergic blocking agents.

### 2.3. Exposure

The exposure variable was log(TG_max/ HDL_min), using the highest TG and lowest HDL values within the first ICU week. This log-transformed ratio was analyzed as both a continuous and a quartile-categorized variable, with the first quartile (Q1) as the reference.

### 2.4. Outcomes

Primary outcomes were 5 mortality end points: in-hospital, ICU, 28-day, 30-day, and 1-year mortality. Secondary outcomes were length of stay (LOS) in the hospital and LOS in the ICU. Mortality data were sourced from hospital and post-discharge records.

### 2.5. Statistical analysis

Descriptive statistics were compared across log(TG_max/ HDL_min) quartiles. Logistic regression estimated odds ratio (OR) with 95% confidence interval (CI) for binary mortality outcomes. Generalized linear models with a Gamma distribution and log link estimated relative length-of-stay ratio (RLR) for LOS. Linear regression on log-transformed LOS (log[LOS + 1]) reported beta coefficients (β) with 95% CI. Four hierarchical regression models were constructed: Model 1 (unadjusted), Model 2 (adjusted for age and sex), Model 3 (Model 2 + BUN_max, creatinine_max, and SOFA score within 24 hours), and Model 4 (Model 3 additionally adjusted for comorbidities [CKD, CHF, CVD, COPD, liver disease, diabetes, cancer, MI] and ICU treatment-related variables, including nephrotoxic drugs, vasopressors, inotropes, diuretics, insulin, glucocorticoids, β-adrenergicblocking agents, MV, and RRT). Because effect estimates of Model 4 were highly consistent with those of Model 3, Model 3 was used as the primary analytical model for restricted cubic spline (RCS) analyses, Receiver operating characteristic (ROC) analysis, subgroup analyses, and sensitivity analyses. RCS regression examined nonlinear associations by plotting predicted probabilities (logistic), RLR (gamma), and fitted values (β, linear) against log(TG_max/ HDL_min). Likelihood ratio tests compared spline vs linear models. ROC analysis with area under the curve (AUC) evaluated the predictive value for 1-year mortality. Residual plots (residuals vs fitted values) were used for linear regression diagnostics. Deviance residuals were examined for logistic and gamma models. Subgroup analyses stratified associations by age (≥65 vs <65 years), sex, CKD, liver disease, diabetes, invasive MV, RRT, nephrotoxic drugs, insulin, glucocorticoids, and SOFA score (≥4 vs <4). Sensitivity analyses were performed by sequentially excluding patients with CKD, liver disease, diabetes, nephrotoxic drug exposure, insulin or steroid use, MV, or RRT, and refitting Model 3 for outcomes that showed statistically significant associations with log(TG_max/HDL_min) in the analysis. Additionally, sensitivity analyses were conducted by excluding patients with a LOS in hospital <7 days and refitting the model for outcomes related to LOS in hospital and log(LOS in hospital + 1). A similar analysis was performed for LOS in ICU and log(LOS in ICU + 1), excluding patients with LOS in hospital <7 days. Results were shown using forest plots with 95% CI and *P*-values for interaction. Two-sided *P*-values <.05 were considered statistically significant.

## 3. Results

### 3.1. Baseline characteristics

**Figure 1.**
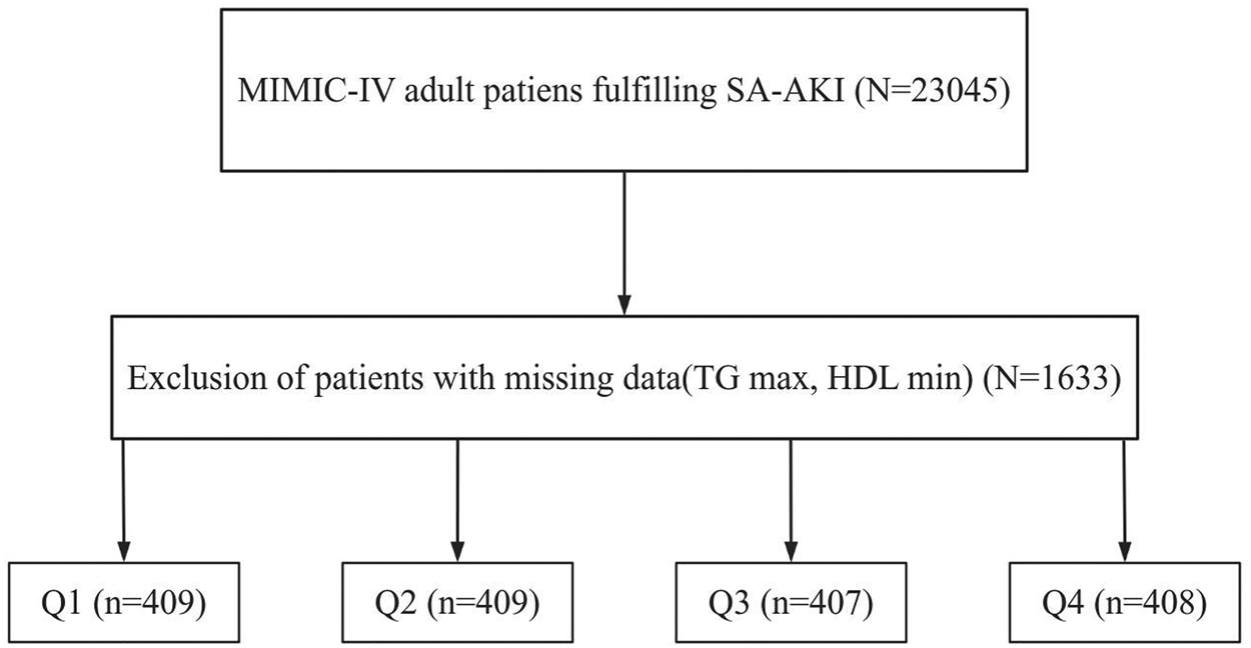
Flow diagram of patient selection. Among 23,045 ICU admissions meeting Sepsis-3 and KDIGO criteria for SA-AKI in the MIMIC-IV database, patients with missing triglyceride or HDL measurements within the first ICU week were excluded, resulting in 1633 patients included in the final analysis. HDL = high-density lipoprotein cholesterol, ICU = intensive care unit, KDIGO = Kidney Disease Improving Global Outcomes, MMIC-IV = Medical Information Mart for Intensive Care IV, SA-AKI = sepsis-associated acute kidney injury, TG = Triglycerides.

Among the 1633 SA-AKI patients, baseline characteristics differed markedly across quartiles of log(TG_max/HDL_min) (Table 1) (Fig. 1). Patients in the fourth quartile (Q4) were younger on average (mean ~59 vs ~70 years in Q1) and exhibited a more pronounced metabolic and inflammatory profile compared to those in the lowest quartile. By definition, Q4 had substantially higher TG levels and lower HDL (median TG ~240 vs 80 mg/dL; HDL ~20 vs 55 mg/dL in Q4 vs Q1). Higher log(TG_max/HDL_min) quartiles were also associated with greater illness severity and organ dysfunction: for example, the median SOFA score doubled from ~4 in Q1 to ~8 in Q4, serum albumin was lower (mean ~3.0 vs 3.6 g/dL), and markers of inflammation or organ injury such as white blood cell count, BUN, creatinine, and liver enzymes (AST/alanine aminotransferase) were all higher in Q4 relative to Q1. These trends indicate that increasing log(TG_max/HDL_min) is associated with progressively worse lipid profiles, more pronounced inflammatory responses, and greater acute illness severity at baseline. Among the 1633 patients with complete TG_max and HDL_min data, all 5 mortality endpoints were fully recorded (missing rate 0%). Consequently, our logistic regression analyses were performed on the entire cohort without the need for data imputation or censoring of follow-up.

**Table 1:** Baseline characteristics of SA-AKI patients stratified by quartiles of log(TG_max/ HDL_min).

Categories	Total (n = 1633)	Q1 (n = 409)	Q2 (n = 409)	Q3 (n = 407)	Q4 (n = 408)	*P*
**Demographic**						
Age, mean ± SD	65.59 ± 15.21	69.92 ± 14.67	68.01 ± 14.42	64.97 ± 14.28	59.42 ± 15.38	<.001
Male, n (%)	957 (58.6%)	215 (52.6%)	239 (58.4%)	238 (58.5%)	265 (65.0%)	.005
Race						.167
White, n (%)	947 (58.0%)	233 (57.0%)	250 (61.1%)	245 (60.2%)	219 (53.7%)	
Black, n (%)	155 (9.5%)	48 (11.7%)	31 (7.6%)	38 (9.3%)	38 (9.3%)	
Others, n (%)	531 (32.5%)	128 (31.3%)	128 (31.3%)	124 (30.5%)	151 (37.0%)	
**Laboratory tests**						
Haemoglobin, g/dl, mean ± SD	11.35 ± 2.10	11.65 ± 2.16	11.69 ± 2.32	11.24 ± 2.39	10.79 ± 2.26	<.001
WBC count, K/µL, M (Q_1_, Q_3_)	12.20 (8.80, 16.40)	11.05 (8.50, 14.20)	11.40 (8.55, 14.95)	12.30 (9.55, 15.88)	12.45 (8.80, 16.90)	<.001
Platelets count, K/µL, M (Q_1_, Q_3_)	181.00 (127.50, 249.00)	198.50 (152.50, 254.50)	196.00 (142.00, 256.62)	196.50 (145.25, 275.75)	179.00 (118.38, 259.62)	.002
Absolute neutrophil count, K/µL, M (Q_1_, Q_3_)	9.39 (6.32, 13.64)	8.45 (5.94, 12.41)	9.12 (6.21, 13.34)	10.18 (6.57, 14.25)	10.07 (6.34, 14.80)	<.001
Absolute lymphocyte count, K/µL, M (Q_1_, Q_3_)	1.09 (0.69, 1.66)	0.97 (0.67, 1.46)	1.09 (0.72, 1.65)	1.21 (0.78, 1.83)	1.10 (0.60, 1.80)	.051
Albumin, g/dl, Mean ± SD	3.08 ± 0.69	3.59 ± 0.57	3.46 ± 0.58	3.29 ± 0.66	3.0 ± 0.66	<.001
Bun, mg/dL, M (Q_1_, Q_3_)	23.00 (15.50, 38.00)	20.00 (14.50, 30.00)	21.00 (15.00, 32.00)	24.50 (16.50, 36.00)	24.50 (17.00, 41.00)	<.001
Creatinine, mg/dL, M (Q_1_, Q_3_)	1.15 (0.80, 1.90)	1.00 (0.75, 1.40)	1.10 (0.85, 1.55)	1.20 (0.93, 1.85)	1.45 (1.00, 2.55)	<.001
Calcium, mg/dL, M (Q_1_, Q_3_)	8.25 (7.80, 8.75)	8.65 (8.20, 9.05)	8.60 (8.20, 8.95)	8.45 (8.04, 8.95)	8.10 (7.60, 8.60)	<.001
Potassium, mEq/L, Mean ± SD	4.34 ± 0.66	4.25 ± 0.64	4.23 ± 0.61	4.34 ± 0.71	4.31 ± 0.69	.899
ALT, U/L, M (Q_1_, Q_3_)	31.00 (17.00, 77.00)	24.00 (16.50, 49.00)	27.00 (16.00, 72.00)	32.25 (17.00, 117.00)	49.50 (22.00, 198.00)	<.001
AST, U/L, M (Q_1_, Q_3_)	49.00 (26.50, 125.50)	36.00 (23.00, 82.62)	39.00 (24.00, 139.75)	56.00 (25.00, 178.00)	97.50 (34.00, 356.75)	<.001
PT, s, M (Q_1_, Q_3_)	14.60 (13.00, 17.70)	13.10 (11.90, 15.00)	13.47 (12.40, 16.15)	14.18 (12.60, 18.55)	14.95 (12.90, 18.34)	<.001
APTT, s, M (Q_1_, Q_3_)	32.40 (28.05, 42.60)	31.00 (27.45, 49.58)	30.80 (26.80, 43.45)	32.38 (27.10, 44.58)	34.00 (28.70, 48.20)	.038
TG, mg/dL, M (Q_1_, Q_3_)	155.00 (98.00, 286.00)	70.00 (58.00, 86.00)	102.00 (86.00, 122.00)	142.00 (114.00, 177.00)	249.00 (174.75, 411.00)	<.001
Cholesterol, mg/dL, M (Q_1_, Q_3_)	130.00 (99.00, 166.00)	144.00 (115.00, 171.00)	130.00 (102.00, 164.00)	133.00 (98.00, 167.00)	123.50 (94.00, 168.00)	<.001
HDL cholesterol, mg/dL, M (Q_1_, Q_3_)	37.00 (26.00, 49.00)	55.00 (46.00, 66.00)	40.00 (34.00, 47.00)	32.00 (26.00, 39.00)	20.00 (13.00, 27.00)	<.001
LDL cholesterol, mg/dL, M (Q_1_, Q_3_)	66.00 (44.00, 93.00)	70.00 (50.00, 91.50)	68.00 (46.00, 95.00)	69.00 (43.00, 98.00)	54.00 (36.00, 82.00)	.001
log(TG_max/ HDL_min)	1.21(0.67, 1.86)	0.32 (0.03, 0.50)	0.93 (0.81, 1.07)	1.50 (1.35, 1.66)	2.45 (2.11, 3.23)	<.001
**Disease scores**						
SOFA 24 h	6.00 (4.00, 9.00)	4.00 (3.00, 6.00)	5.00 (3.00, 7.00)	5.00 (3.00, 8.00)	8.00 (5.00, 10.00)	<.001
**AKI stage**						.226
I, n (%)	21299 (92.4%)	353 (86.3%)	368 (90.0%)	363 (89.2%)	370 (90.7%)	
II, n (%)	1285 (5.6%)	43 (10.5%)	29 (7.1%)	34 (8.4%)	23 (5.6%)	
III, n (%)	461 (2.0%)	13 (3.2%)	12 (2.9%)	10 (2.5%)	15 (3.7%)	
**Comorbidities**						
COPD, n (%)	305 (18.7%)	79 (19.3%)	73 (17.8%)	80 (19.7%)	73 (17.9%)	.874
CHF, n (%)	611 (37.4%)	164 (40.1%)	159 (38.9%)	163 (40.0%)	125 (30.6%)	.014
Diabetes, n (%)	571 (35.0%)	109 (26.7%)	148 (36.2%)	152 (37.3%)	162 (39.7%)	<.001
CVD, n (%)	766 (46.9%)	232 (56.7%)	211 (51.6%)	198 (48.6%)	125 (30.6%)	<.001
Cancer, n (%)	231 (14.1%)	38 (9.3%)	55 (13.4%)	62 (15.2%)	76 (18.6%)	.002
Liver disease, n (%)	420 (25.7%)	63 (15.4%)	84 (20.5%)	118 (29.0%)	155 (38.0%)	<.001
CKD, n (%)	398 (24.4%)	86 (21.0%)	93 (22.7%)	114 (28.0%)	105 (25.7%)	.092
MI, n (%)	369 (22.6%)	103 (25.2%)	90 (22.0%)	99 (24.3%)	77 (18.9%)	.139
**Drug use**						
Nephrotoxins, n (%)	806 (49.4%)	153 (37.4%)	191 (46.7%)	216 (53.1%)	246 (60.3%)	<.001
Diuretics, n (%)	623 (38.2%)	136 (33.3%)	158 (38.6%)	172 (42.3%)	157 (38.5%)	.14
Vasopressors, n (%)	744 (45.6%)	147 (35.9%)	165 (40.3%)	205 (50.4%)	227 (55.6%)	<.001
Insulin, n (%)	1128 (69.1%)	273 (66.7%)	284 (69.4%)	291 (71.5%)	280 (68.6%)	.61
Inotropes, n (%)	91 (5.6%)	19 (4.6%)	20 (4.9%)	26 (6.4%)	26 (6.4%)	.39
Glucocorticoids, n (%)	326 (20.0%)	61 (14.9%)	81 (19.8%)	90 (22.1%)	94 (23.0%)	.006
β-adrenergic blocking agents, n (%)	735 (45.0%)	226 (55.3%)	195 (47.7%)	186 (45.7%)	128 (31.4%)	<.001
**Therapies**						
RRT, n (%)	110 (6.7%)	15 (3.7%)	21 (5.1%)	22 (5.4%)	52 (12.7%)	<.001
MV, n (%)	718 (44.0%)	161 (39.4%)	168 (41.1%)	187 (45.9%)	202 (49.5%)	
**Outcomes**						
In-hospital mortality, n(%)	363 (22.2%)	83 (20.3%)	90 (22.0%)	96 (23.6%)	94 (23.0%)	.685
ICU mortality, n (%)	275 (16.8%)	66 (16.1%)	64 (15.6%)	73 (17.9%)	72 (17.6%)	.777
28-day mortality, n (%)	421 (25.8%)	105 (25.7%)	110 (26.9%)	109 (26.8%)	97 (23.8%)	.72
30-day mortality, n (%)	425 (26.0%)	107 (26.2%)	110 (26.9%)	109 (26.8%)	99 (24.3%)	.815
1-yr mortality, n (%)	662 (40.5%)	161 (39.4%)	174 (42.5%)	165 (40.5%)	162 (39.7%)	.793
LOS in hospital, M (Q_1_, Q_3_)	12.18 (7.30, 20.82)	10.35 (6.51, 16.66)	11.64 (6.96, 19.35)	12.79 (7.17, 22.20)	15.01 (9.10, 27.07)	<.001
LOS in ICU, M (Q_1_, Q_3_)	5.29 (2.86, 10.64)	5.29 (2.81, 10.06)	4.75 (2.75, 9.72)	5.37 (2.79, 10.15)	5.95 (3.14, 12.33)	<.001

AKI = acute kidney injury, ALT = alanine aminotransferase, APTT = activated partial thromboplastin time, AST = aspartate aminotransferase, BUN = blood urea nitrogen, CHF = congestive heart failure, CKD = chronic kidney disease, COPD = chronic obstructive pulmonary disease, CVD = cardiovascular disease, HDL = high-density lipoprotein cholesterol, ICU = intensive care unit, LDL = low-density lipoprotein cholesterol, LOS = length of stay, M = Median, MI = myocardial infarction, MV = mechanical ventilation, PT = prothrombin time, Q1 = first quartile, Q2 = second quartile, Q3 = third quartile, Q4 = fourth quartile, RRT = renal replacement therapy, SD = standard deviation, SOFA = Sequential Organ Failure Assessment, TG = triglycerides, WBC = white blood cell.

### 3.2. Logistic regression

Logistic regression analyses demonstrated a positive association between log(TG_max/HDL_min) and mortality risk, with the strength of association varying by outcome and adjustment model (Table 2). Treating log(TG_max/HDL_min) as a continuous predictor, higher values were linked to increased odds of mortality in several endpoints. For in-hospital mortality, each 1-unit increment in log(TG_max/HDL_min) was associated with a nonsignificant trend toward higher risk in the univariate model (OR 1.11, 95% CI 1.00–1.24; *P* = .0547), which became significant after adjusting for age and sex (Model 2: OR 1.20, 95% CI 1.07–1.35; *P* = .0015). However, this association attenuated in the fully adjusted model including age, sex, BUN_max, creatinine_max, and SOFA (Model 3: OR 1.10, 95% CI 0.97–1.24; *P* = .1241). A similar pattern was observed for ICU mortality (Model 2 OR 1.14, 95% CI 1.00–1.29; *P* = .0468, becoming nonsignificant in Model 3 with *P* = .6137). Neither 28-day nor 30-day mortality showed significant independent associations with continuous log(TG_max/HDL_min) in the fully adjusted analysis (*P* > .5 for both). In contrast, 1-year mortality exhibited the strongest and most consistent relationship: in the age- and sex-adjusted model, each unit increase in log(TG_max/HDL_min) conferred a 24% higher odds of 1-year mortality (OR 1.24, 95% CI 1.12–1.37; *P* < .001), and this association remained statistically significant even after full multivariable adjustment (Model 3 OR 1.15, 95% CI 1.03–1.28; *P* = .0107).

**Table 2:** Association between log(TG_max/ HDL_min) and mortality outcomes across different adjustment models (Model 1–4).

Categories	Model 1		Model 2		Model 3		Model 4	
	OR (95% CI)	*P*-value	OR (95% CI)	*P*-value	OR (95% CI)	*P*-value	OR (95% CI)	*P*-value
**In-hospital mortality**								
log(TG_max/ HDL_min), continuous	1.11 (1.00–1.24)	.0547	1.20 (1.07–1.35)	.0015	1.10 (0.97–1.24)	.1241	1.12 (0.98–1.27)	.0911
Q1	1.00 (Reference)	NA	1.00 (Reference)	NA	1.00 (Reference)	NA	1.00 (Reference)	NA
Q2	1.11 (0.79–1.55)	.5490	1.14 (0.81–1.60)	.4576	1.06 (0.75–1.49)	.7489	1.11 (0.78–1.58)	.5703
Q3	1.21 (0.87–1.69)	.2560	1.32 (0.94–1.86)	.1030	1.15 (0.82–1.63)	.4158	1.12 (0.78–1.61)	.5234
Q4	1.18 (0.84–1.64)	.3411	1.40 (0.99–1.98)	.0571	1.04 (0.72–1.49)	.8477	1.11 (0.75–1.63)	.5962
*P* for trend	1.08 (0.98–1.19)	.1130	1.13 (0.987–1.301)	.0760	1.08 (0.92–1.28)	.3460	1.03 (0.92–1.17)	.5952
**ICU mortality**								
log(TG_max/ HDL_min), continuous	1.10 (0.98–1.24)	.1124	1.14 (1.00–1.29)	.0468	1.04 (0.91–1.18)	.6137	1.04 (0.90–1.20)	.5893
Q1	1.00 (Reference)	NA	1.00 (Reference)	NA	1.00 (Reference)	NA	1.00 (Reference)	NA
Q2	0.96 (0.66–1.40)	.8483	0.96 (0.66–1.40)	.8464	0.89 (0.61–1.30)	.5434	0.92 (0.62–1.37)	.6941
Q3	1.14 (0.79–1.64)	.4944	1.17 (0.81–1.69)	.4074	1.01 (0.69–1.48)	.9511	0.96 (0.65–1.42)	.8349
Q4	1.11 (0.77–1.61)	.5647	1.18 (0.81–1.72)	.3956	0.86 (0.58–1.29)	.4757	0.90 (0.59–1.38)	.628
*P* for trend	1.18 (1.02–1.36)	.0220	1.18 (1.03–1.36)	.0210	1.18 (1.03–1.37)	.0210	0.97 (0.85–1.11)	0.6917
**28-day mortality**								
log(TG_max/ HDL_min), continuous	0.99 (0.89–1.10)	.8585	1.12 (1.00–1.25)	.0577	1.03 (0.91–1.16)	.6839	1.05 (0.93–1.20)	.4235
Q1	1.00 (Reference)	NA	1.00 (Reference)	NA	1.00 (Reference)	NA	1.00 (Reference)	NA
Q2	1.07 (0.78–1.45)	.6913	1.12 (0.82–1.54)	.4823	1.06 (0.77–1.46)	.7339	1.11 (0.80–1.55)	.5259
Q3	1.06 (0.78–1.45)	.7188	1.22 (0.89–1.68)	.2175	1.08 (0.78–1.50)	.6422	1.07 (0.76–1.51)	.6861
Q4	0.90 (0.66–1.24)	.5296	1.20 (0.86–1.68)	.2868	0.92 (0.64–1.30)	.6278	1.01 (0.70–1.47)	.9478
*P* for trend	1.19 (1.03–1.38)	.0180	1.19 (1.0–1.38)	.0180	1.2 (1.03–1.39)	.0160	1.00 (0.89–1.13)	.9783
**30-day mortality**								
log(TG_max/ HDL_min), continuous	0.99 (0.89–1.10)	.8230	1.12 (1.00–1.25)	.0546	1.03 (0.91–1.16)	.6530	1.06 (0.93–1.20)	.384
Q1	1.00 (Reference)	NA	1.00 (Reference)	NA	1.00 (Reference)	NA	1.00 (Reference)	NA
Q2	1.04 (0.76–1.42)	.8122	1.09 (0.80–1.50)	.5771	1.03 (0.75–1.42)	.8357	1.09 (0.78–1.52)	.5963
Q3	1.03 (0.76–1.41)	.8409	1.20 (0.87–1.65)	.2683	1.06 (0.77–1.47)	.7255	1.06 (0.75–1.49)	.7559
Q4	0.90 (0.66–1.24)	.5326	1.21 (0.87–1.69)	.2541	0.93 (0.66–1.32)	.7005	1.04 (0.72–1.50)	.8419
*P* for trend	1.04 (0.9–1.2)	.6100	1.04 (0.9–1.2)	.6010	1.04 (0.9–1.21)	.5790	1.01 (0.90–1.13)	.8855
**1-yr mortality**								
log(TG_max/ HDL_min), continuous	1.05 (0.96–1.15)	.3151	1.24 (1.12–1.37)	<**.0001**	1.15 (1.03–1.28)	.0107	1.16 (1.03–1.30)	.0133
Q1	1.00 (Reference)	NA	1.00 (Reference)	NA	1.00 (Reference)	NA	1.00 (Reference)	NA
Q2	1.14 (0.86–1.51)	.3554	1.23 (0.93–1.65)	.1522	1.19 (0.89–1.59)	.2511	1.20 (0.89–1.63)	.2341
Q3	1.05 (0.79–1.39)	.7316	1.27 (0.95–1.70)	.1074	1.15 (0.85–1.54)	.3655	1.09 (0.80–1.48)	.6007
Q4	1.01 (0.77–1.34)	.9205	1.49 (1.10–2.02)	.0092	1.18 (0.86–1.62)	.3011	1.19 (0.85–1.67)	.2988
*P* for trend	1.17 (1.01–1.35)	.0310	1.17 (1.02–1.35)	.0290	1.17 (1.02–1.36)	.0280	1.04 (0.94–1.16)	.4303

Model 1 (unadjusted); Model 2 (adjusted for age and sex); Model 3 (Model 2 + BUN_max, serum creatinine_max, and SOFA score); and Model 4 (Model 3 + comorbidities and treatment-related variables, including CKD, CHF, CVD, COPD, liver disease, diabetes, cancer, MI, nephrotoxic drugs, vasopressors, inotropes, diuretics, insulin, glucocorticoids, β-adrenergic blocking agents, MV, and RRT).

CI = confidence interval, HDL = high-density lipoprotein cholesterol, ICU = intensive care unit, NA = not applicable, OR = odds ratio, Q1 = first quartile, Q2 = second quartile, Q3 = third quartile, Q4 = fourth quartile, TG = triglycerides.

Analyses using quartiles of log(TG_max/HDL_min) yielded consistent findings. Patients in the highest quartile (Q4) generally had greater mortality odds than those in the lowest quartile Q1, with a clear dose-response trend across increasing quartiles for several outcomes. Notably, for 1-year mortality, the Q4 group had a significantly higher risk compared to Q1 in Model 2 (OR 1.49, 95% CI 1.10–2.02; *P* = .0092), an effect that was attenuated in Model 3 (OR 1.18, 95% CI 0.86–1.62; *P* = .3011) but still reflected in a significant linear trend across quartiles (*P* for trend = .0280). ICU and 28-day mortality also demonstrated significant graded relationships: for example, even though the extreme quartile comparisons were not individually significant, there was a significant increasing trend in ICU mortality risk from Q1 to Q4 in the fully adjusted model (*P* for trend = .0210), and a similar trend was observed for 28-day mortality (*P* for trend = .0160 in Model 3). In contrast, in-hospital mortality showed only a borderline quartile effect (e.g., Q4 vs Q1 OR ~1.4 in Model 2, *P* = .0571) that did not reach significance after full adjustment (*P* for trend = .346), and no significant quartile association was seen for 30-day mortality (*P* for trend > .5).

In summary, elevated log(TG_max/HDL_min) was most strongly and consistently associated with increased 1-year mortality, while associations with shorter-term mortality end points were weaker and often lost significance after multivariable adjustment.

### 3.3. Gamma regression

#### 3.3.1. LOS in hospital

When log(TG_max/HDL_min) was treated as a continuous covariate, a one-unit increase was associated with an 18 % longer LOS in hospital in the univariate model (Model 1, RLR = 1.18, 95 % CI 1.13–1.22; *P* < .0001) (Table 3). The effect was attenuated and lost nominal significance after adjustment for age and sex (Model 2, RLR = 1.06, 0.98–1.15; *P* = .12) but reemerged when BUN_max, creatinine_max and SOFA were additionally included (Model 3, RLR = 1.13, 1.08–1.18; *P* < .0001). Categorical analyses showed a clear dose-response: compared with Q1, patients in the third quartile (Q3) and Q4 had 20 % (RLR = 1.20, 1.07–1.35) and 34 % (RLR = 1.34, 1.19–1.51) longer stays, respectively, in the fully adjusted model, whereas the second quartile (Q2) did not differ from reference. Linear trend testing across quartiles remained highly significant in every model (*P* for trend < .0001), confirming a monotonic increase in hospital LOS with higher log(TG_max/HDL_min).

**Table 3:** RLR associated with log(TG_max/ HDL_min) for LOS in hospital and ICU: gamma regression models (Model 1–4).

Categories	Model 1		Model 2		Model 3		Model 4	
Outcomes	RLR (95% CI)	*P*-value	RLR (95% CI)	*P*-value	RLR (95% CI)	*P*-value	RLR (95% CI)	*P*-value
**LOS in hospital**								
log(TG_max/ HDL_min), continuous	1.18 (1.13–1.22)	<.0001	1.06 (0.98–1.15)	.1238	1.13 (1.08–1.18)	<.0001	1.13 (1.09–1.18)	<.0001
Q1	1.00 (Reference)	NA	1.00 (Reference)	NA	1.00 (Reference)	NA	1.00 (Reference)	NA
Q2	1.10 (0.98–1.24)	.09	1.08 (0.96–1.21)	.1928	1.07 (0.95–1.20)	.2429	1.07 (0.95–1.19)	.257
Q3	1.28 (1.14–1.44)	<.0001	1.22 (1.09–1.37)	.0008	1.20 (1.07–1.35)	.0019	1.17 (1.05–1.31)	.0055
Q4	1.51 (1.35–1.70)	<.0001	1.37 (1.22–1.54)	<.0001	1.34 (1.19–1.51)	<.0001	1.36 (1.20–1.53)	<.0001
P for trend	1.15 (1.11–1.19)	<.0001	1.11 (1.07–1.15)	<.0001	1.10 (1.06–1.15)	<.0001	1.11 (1.06–1.15)	<.0001
**LOS in ICU**								
log(TG_max/ HDL_min), continuous	1.13 (1.08–1.18)	<.0001	1.11 (1.06–1.16)	<.0001	1.12 (1.07–1.18)	<.0001	1.12 (1.07–1.18)	<.0001
Q1	1.00 (Reference)	NA	1.00 (Reference)	NA	1.00 (Reference)	NA	1.00 (Reference)	NA
Q2	0.97 (0.85–1.11)	.6481	0.96 (0.84–1.10)	.5443	0.96 (0.84–1.10)	.5611	0.97 (0.86–1.10)	.6817
Q3	1.05 (0.92–1.20)	.4927	1.02 (0.89–1.17)	.7633	1.04 (0.90–1.19)	.6056	1.02 (0.90–1.16)	.745
Q4	1.28 (1.12–1.46)	.0003	1.20 (1.05–1.38)	.0093	1.24 (1.07–1.43)	.0034	1.28 (1.12–1.47)	.0003
*P* for trend	1.08 (1.04–1.13)	.0002	1.06 (1.02–1.11)	.0070	1.07 (1.02–1.12)	.0029	1.08 (1.03–1.13)	.0004

Model 1 (unadjusted); Model 2 (adjusted for age and sex); Model 3 (Model 2 + BUN_max, serum creatinine_max, and SOFA score); and Model 4 (Model 3 + comorbidities and treatment-related variables, including CKD, CHF, CVD, COPD, liver disease, diabetes, cancer, MI, nephrotoxic drugs, vasopressors, inotropes, diuretics, insulin, glucocorticoids, β-adrenergic blocking agents, MV, and RRT).

CI = confidence interval, HDL = high-density lipoprotein cholesterol, ICU = intensive care unit, LOS = length of stay, NA = not applicable, Q1 = first quartile, Q2 = second quartile, Q3 = third quartile, Q4 = fourth quartile, RLR = relative length-of-stay ratio, TG = triglycerides.

#### 3.3.2. LOS in ICU

For LOS in ICU, the continuous log(TG_max/HDL_min) term was consistently significant across all specifications (Model 1 RLR = 1.13, 1.08–1.18; Model 2 RLR = 1.11, 1.06–1.16; Model 3 RLR = 1.12, 1.07–1.18; all *P* < .0001), indicating roughly a 12 % prolongation of ICU days per unit increase (Table 3). Quartile analysis again highlighted the extremes: only the highest quartile (Q4) showed a significant excess LOS after full adjustment (RLR = 1.24, 1.07–1.43; *P* = .0034), whereas Q2 to Q3 remained neutral. Nevertheless, the overall linear trend from Q1 to Q4 persisted (Model 3 *P* for trend = .0029).

Taken together, higher log(TG_max/HDL_min) values: particularly those in the upper quartile: were independently associated with progressively longer hospital and ICU stays, even after controlling for key renal and illness severity covariates.

### 3.4. Linear regression

#### 3.4.1. LOS in hospital (log[LOS in hospital+1])

In univariate analysis (Model 1) each one-unit increase in log(TG_max/HDL_min) corresponded to a β coefficient of 0.14 (95 % CI 0.11–0.18; *P* < .0001), indicating a 15% to 20% longer hospitalization after back-transformation (Table 4). The association persisted after adjustment for age and sex (Model 2 β = 0.11, 0.08–0.15; *P* < .0001) and remained unchanged when BUN_max, creatinine_max and SOFA were added (Model 3 β = 0.11, 0.08–0.15; *P* < .0001). Categorical analyses showed a clear dose-response: compared with the lowest quartile, patients in Q3 (β = 0.12, 0.02–0.22; *P* = .0242) and Q4 (β = 0.26, 0.15–0.36; *P* < .0001) experienced significantly longer stays in the fully adjusted model, whereas Q2 remained neutral. The linear trend across quartiles was highly significant in every model (*P* for trend < .0001), confirming a monotonic increase in hospital LOS with rising log(TG_max/HDL_min).

**Table 4:** Association between log(TG_max/ HDL_min) and log(LOS + 1) in hospital and ICU: linear regression (Model 1–4).

Categories	Model 1		Model 2		Model 3		Model 4	
	β (95% CI)	*P*-value	β (95% CI)	*P*-value	β (95% CI)	*P*-value	β (95% CI)	*P*-value
**log(LOS in hospital + 1**)								
log(TG_max/ HDL_min), continuous	0.14 (0.11–0.18)	<.0001	0.11 (0.08–0.15)	<.0001	0.11 (0.08–0.15)	<.0001	0.11 (0.08–0.15)	<.0001
Q1	1.00 (Reference)	NA	1.00 (Reference)	NA	1.00 (Reference)	NA	1.00 (Reference)	NA
Q2	0.08 (-0.02–0.18)	.1070	0.07 (-0.03–0.16)	.1934	0.06 (-0.04–0.16)	.2100	0.06 (-0.04–0.15)	.2443
Q3	0.16 (0.06–0.26)	.0017	0.12 (0.02–0.22)	.0195	0.12 (0.02–0.22)	.0242	0.10 (-0.00–0.19)	.0601
Q4	0.35 (0.25–0.45)	<.0001	0.26 (0.16–0.36)	<.0001	0.26 (0.15–0.36)	<.0001	0.28 (0.17–0.38)	<.0001
*P* for trend	0.11 (0.08–0.14)	<.0001	0.08 (0.05–0.12)	<.0001	0.08 (0.05–0.12)	<.0001	0.09 (0.05–0.12)	<.0001
**log(LOS in ICU + 1**)								
log(TG_max/ HDL_min), continuous	0.10 (0.06–0.13)	<.0001	0.08 (0.04–0.11)	<.0001	0.09 (0.05–0.12)	<.0001	0.10 (0.06–0.13)	<.0001
Q1	1.00 (Reference)	NA	1.00 (Reference)	NA	1.00 (Reference)	NA	1.00 (Reference)	NA
Q2	-0.04 (-0.15–0.06)	.3841	-0.06 (-0.16–0.04)	.2590	-0.05 (-0.15–0.05)	.287	-0.03 (-0.12–0.06)	.5240
Q3	0.00 (-0.10–0.11)	.9285	-0.02 (-0.12–0.08)	.6625	-0.01 (-0.11–0.09)	.8749	-0.01 (-0.10–0.09)	.9095
Q4	0.15 (0.05–0.25)	.0031	0.09 (-0.01–0.20)	.0725	0.12 (0.01–0.23)	.0300	0.18 (0.08–0.28)	.0004
*P* for trend	0.05 (0.02–0.08)	.0019	0.03 (-0.00–0.06)	.0613	0.04 (0.00–0.07)	.0251	0.06 (0.02–0.09)	.0007

Model 1 (unadjusted); Model 2 (adjusted for age and sex); Model 3 (Model 2 + BUN_max, serum creatinine_max, and SOFA score); and Model 4 (Model 3 + comorbidities and treatment-related variables, including CKD, CHF, CVD, COPD, liver disease, diabetes, cancer, MI, nephrotoxic drugs, vasopressors, inotropes, diuretics, insulin, glucocorticoids, β-adrenergic blocking agents, MV, and RRT).

Β = β coefficient, CI = confidence interval, HDL = high-density lipoprotein cholesterol, ICU = intensive care unit, LOS = length of stay, NA = not applicable, Q1 = first quartile, Q2 = second quartile, Q3 = third quartile, Q4 = fourth quartile, TG = triglycerides.

#### 3.4.2. LOS in ICU (log [LOS in ICU+1])

A similar but slightly weaker pattern emerged for LOS in the ICU. The continuous predictor was significant in all models (Model 3 β = 0.09, 0.05–0.12; *P* < .0001) (Table 4). Quartile analysis showed that only the highest quartile retained significance after full adjustment (Q4 vs Q1 β = 0.12, 0.01–0.23; *P* = .0300), while Q2 to Q3 were nonsignificant. Nonetheless, the overall graded relationship remained evident (Model 3 *P* for trend = .0251).

Collectively, these linear regression findings indicate that elevated log(TG_max/HDL_min), especially values in the upper quartile, is independently associated with progressively longer hospital and ICU stays even after accounting for demographic factors, renal function, and illness severity.

### 3.5. Robustness assessment using extended adjustment (model 4)

To further evaluate the robustness of the associations, we constructed an extended multivariable model (Model 4) that additionally incorporated a broad set of comorbidities and ICU treatment-related variables (including CKD, CHF, CVD, COPD, liver disease, diabetes, cancer, MI, nephrotoxic drugs, vasopressors, inotropes, diuretics, insulin, glucocorticoids, β-adrenergic blocking agents, MV, and RRT). Across logistic, gamma, and linear regression analyses, the effect estimates for log(TG_max/HDL_min), in both continuous and quartile-based forms, remained highly consistent with those observed in Model 3. Neither the magnitude nor the direction of associations showed material attenuation or reversal after this broader adjustment. These findings indicate that further controlling for comorbidities and treatment exposures did not materially alter the observed relationships, confirming that Model 3 already achieved adequate confounding adjustment and can be considered the primary analytical model.

### 3.6. Model diagnostics

Visual inspection of deviance-residual plots for all 5 mortality models (Model 3) showed an even, symmetric scatter around their respective reference lines, with no discernible patterns or extreme leverage points, indicating good fit and no major departure from model assumptions (Supplementary Figure S1A,S1B,S1C,S1D and S1E, Supplemental Digital Content 1). Similarly, deviance-residual plots for the gamma regressions of LOS in hospital and ICU, as well as the residual-versus-fitted plots for the linear models of log-transformed LOS, displayed homoscedastic, roughly centered clouds without funneling or curvature (Supplementary Figure S1F,S1G,S1H,S1I, Supplemental Digital Content 2). Taken together, these diagnostics suggest that the specification of covariates, link functions, and distributional choices was appropriate and that the reported effect estimates are not unduly influenced by outliers or systematic lack of fit.

### 3.7. RCS analysis

**Figure 2.**
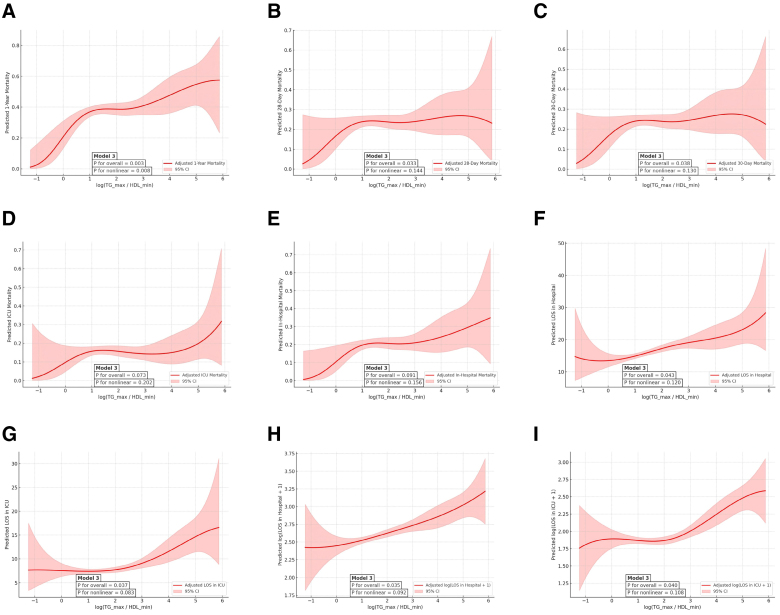
RCS analyses of fully adjusted (Model 3) associations between log(TG_max/HDL_min) and clinical outcomes, showing predicted values on the y-axis. (A–E) Estimated probability of 1-year (A), 28-day (B), 30-day (C), ICU (D) and in-hospital (E) mortality from multivariable logistic models. (F–G) Predicted mean length of stay in hospital (F) and ICU (G) from multivariable gamma regression with log link. (H–I) Predicted expected log-transformed LOS + 1 in hospital (H) and ICU (I) from multivariable linear regression. Shaded areas are 95% confidence intervals. ICU = intensive care unit.

The fully adjusted splines (c) confirmed a broadly monotonic, upward relationship between log(TG_max/ HDL_min) and all clinical end points, but only 1-year mortality displayed clear non-linearity. For this outcome the curve rose steeply from the lowest values, flattened around log-ratio ≈ 1 to 2, and ascended again beyond ≈ 3, with both the overall (*P* = .003) and nonlinear components (*P* = .008) reaching significance (Fig. 2A). By contrast, 28- and 30-day mortality showed modest linear increases (overall *P* ≤ .04) but no evidence of non-linearity (*P* > .13), while in-hospital and ICU mortality curves were visually similar yet did not meet the overall significance threshold (overall *P* = .091 and 0.073, respectively) (Figs. 2B-E).

For LOS outcomes, the splines from both the log-transformed linear models and the gamma models indicated steadily longer stays with higher log(TG_max/ HDL_min) (Figs. 2F-I). The associations were significant overall for LOS in hospital (*P* = .035 for log-transformed; 0.043 for gamma) and LOS in ICU (*P* = .040 and 0.037, respectively), whereas the nonlinear terms remained nonsignificant across all LOS analyses.

Collectively, these RCS results suggest that the risk elevation is essentially linear for short-term mortality and length-of-stay, but becomes J-shaped and more pronounced for long-term (1-year) mortality at both lower and especially higher extremes of the log(TG_max/HDL_min).

### 3.8. ROC curve analysis

**Figure 3.**
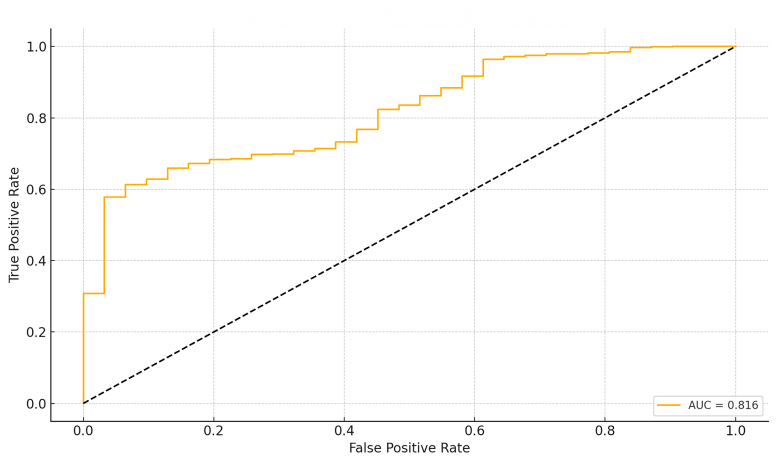
ROC curve for the fully adjusted multivariable logistic regression model (Model 3) predicting 1-year mortality. The plot displays the true positive rate (sensitivity) versus the false positive rate (1: specificity) across all thresholds; the solid line represents the model’s performance (AUC = 0.816), and the dashed diagonal indicates no-discrimination (AUC = 0.5). AUC = area under the curve.

To evaluate the discriminative performance of the multivariable logistic regression model (Model 3) for predicting 1-year mortality, we constructed an ROC curve based on the predicted probabilities. The model, which included log(TG_max/ HDL_min) along with age, gender, BUN_max, creatinine_max, and SOFA score within 24 hours, demonstrated a good predictive ability, with an AUC of 0.816 (Fig. 3), indicating acceptable discrimination within this cohort. These results suggest that the log(TG_max/ HDL_min) may provide additional information for risk assessment in patients with SA-AKI. However, as this analysis was exploratory and no internal validation was performed, the predictive performance should be interpreted with caution and requires confirmation in future studies.

### 3.9. Subgroup analysis

#### 3.9.1. Mortality outcomes

**Figure 4.**
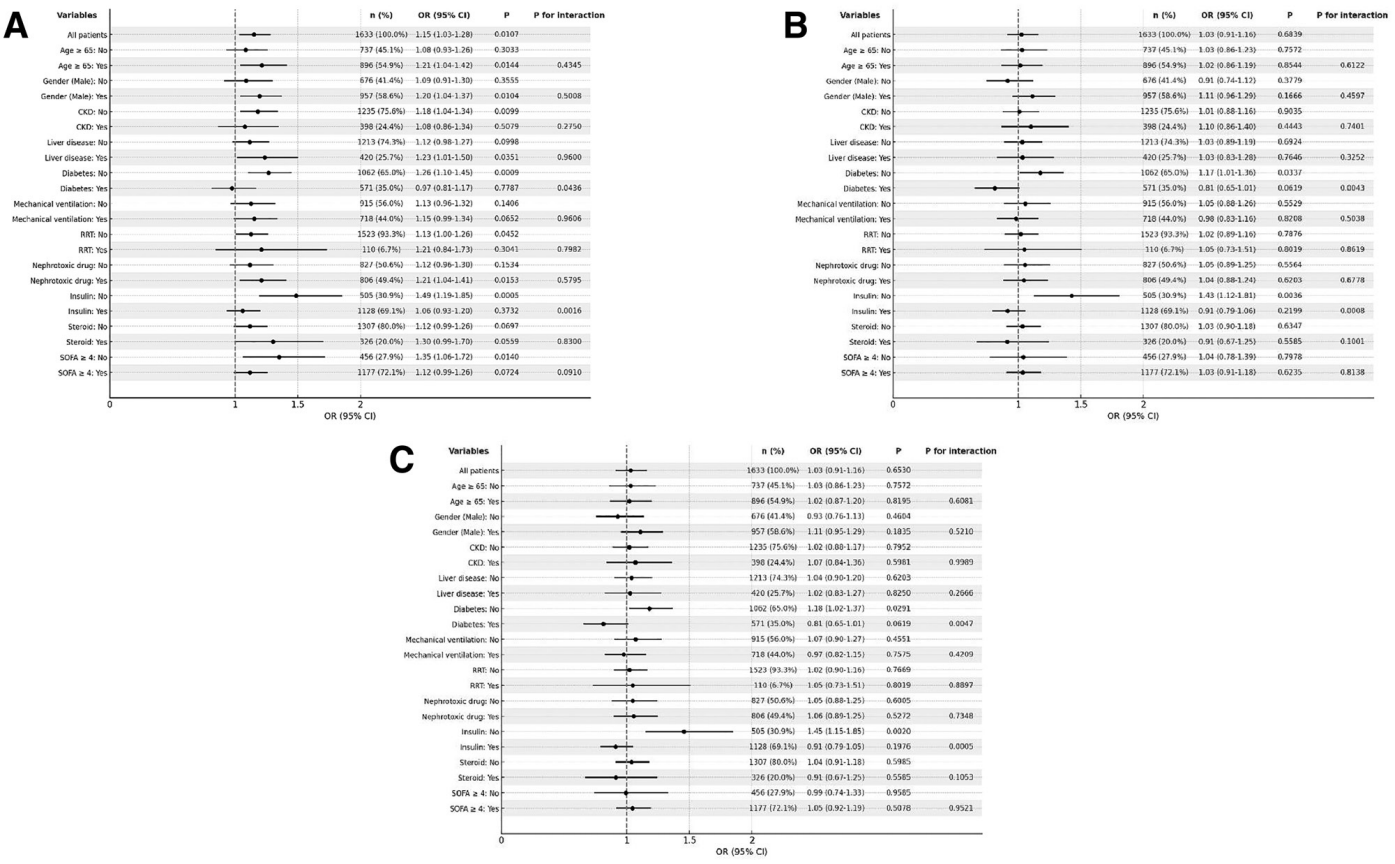
Forest‐plot subgroup analyses of the associations between log(TG_max/HDL_min) and selected mortality outcomes in the fully adjusted model (Model 3). Panels show adjusted OR with 95% CI for 1-year (A), 28-day (B), and 30-day (C) mortality across prespecified subgroups. CI = confidence interval, OR = odds ratio.

**Figure 5.**
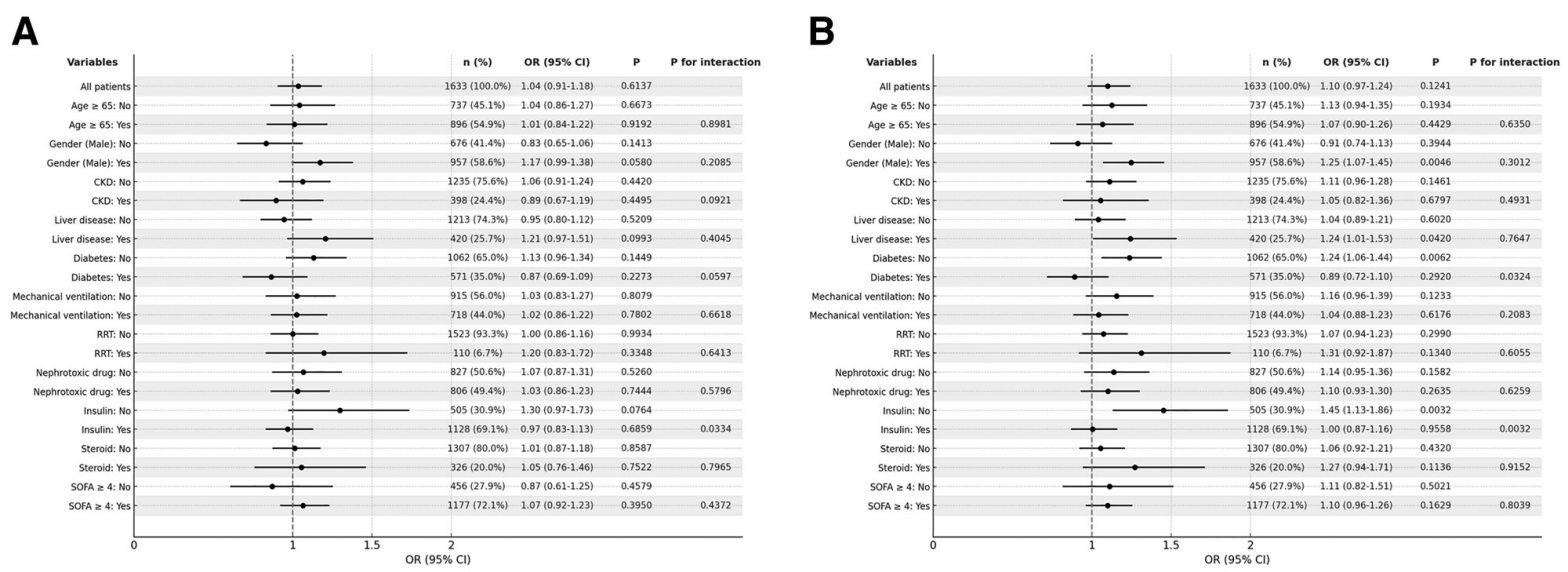
Forest‐plot subgroup analyses of the associations between log(TG_max/HDL_min) and additional mortality outcomes in the fully adjusted model (Model 3). Panels show adjusted OR with 95% CI for ICU (A) and in-hospital (B) mortality across prespecified subgroups. ICU = intensive care unit, OR = odds ratio.

In fully adjusted Model 3, log(TG_max/ HDL_min) was a significant independent predictor of 1-year mortality (OR ≈ 1.15, 95%CI 1.03–1.28, P ≈ 0.01) (Fig. 4A). Shorter-term mortality end points (28-day, 30-day, ICU, in-hospital) showed no significant overall effect, but subgroup analysis revealed that patients without diabetes had consistently higher odds of death associated with the factor, whereas diabetic patients did not (Figure 4B-C, Figure 5). For example, in non-diabetics the 1-year mortality OR was 1.26 (95%CI 1.10–1.45, *P* = .0009) and in-hospital mortality OR 1.24 (1.06–1.44, *P* = .0062), with similar elevated odds at 28- and 30-day mortality (~1.17, *P* < .05). In contrast, diabetics showed no increase (1-year OR ≈ 0.97, *P* = .78; in-hospital OR ≈ 0.89, *P* = .29), and the interaction by diabetes was significant (*P* < .05 for 1-year, in-hospital, and 28/30-day mortality). Insulin use showed a similar pattern: patients not on insulin had higher mortality (e.g., 1-year OR 1.49 (1.19–1.85), *P* = .0005; 28-day OR 1.43, *P* = .0036; 30-day OR 1.45, *P* = .0020; in-hospital OR 1.45, *P* = .0032), whereas those receiving insulin had no significant risk increase (OR ~1.0, *P* > .1), with interaction *P* = .0016 (1-year) and ~0.0005 to 0.003 for the other mortality end points. No other subgroup factor (age, sex, CKD, liver disease, MV, RRT, nephrotoxic drugs, steroid use, high SOFA, etc) showed a significant interaction effect on mortality, indicating that the mortality impact of this factor was most pronounced specifically in non-diabetic patients not on insulin therapy.

#### 3.9.2. LOS outcomes

**Figure 6.**
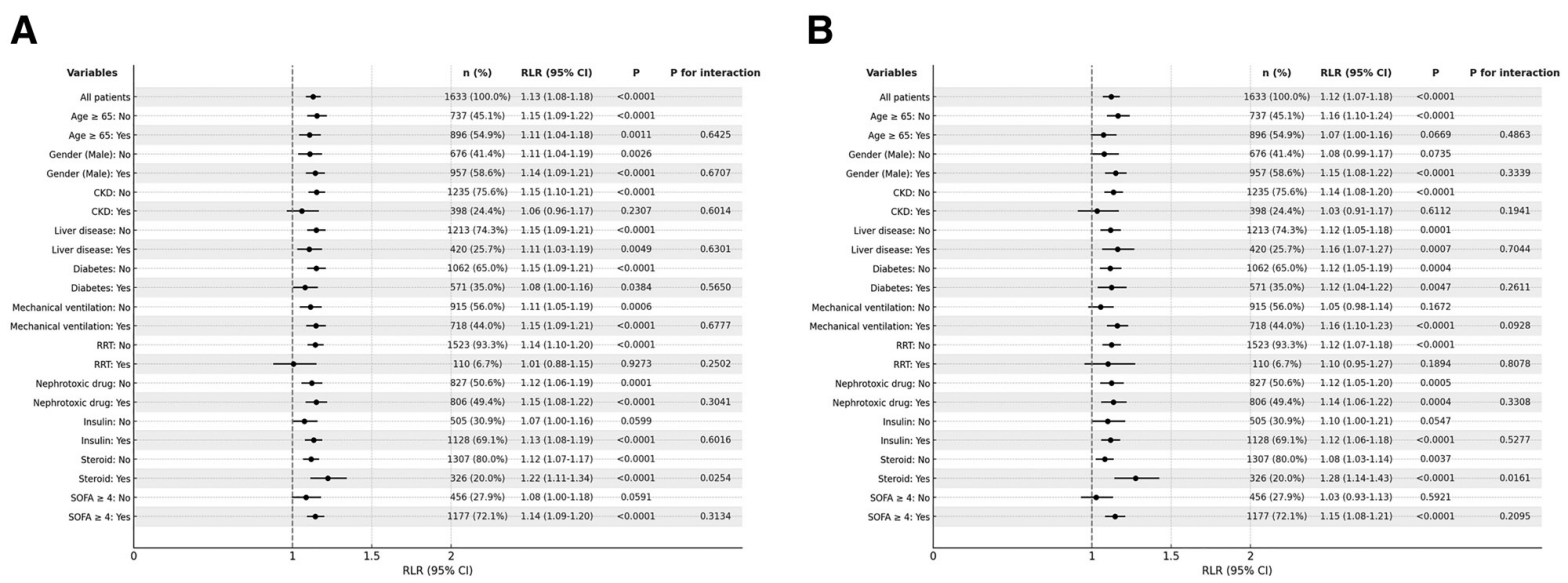
Forest‐plot subgroup analyses of the associations between log(TG_max/HDL_min) and length-of-stay outcomes based on gamma regression models (Model 3). Panels show adjusted RLR with 95% CI for LOS in hospital (A) and LOS in ICU (B) across prespecified subgroups. ICU = intensive care unit, RLR = Relative length-of-stay ratio.

**Figure 7.**
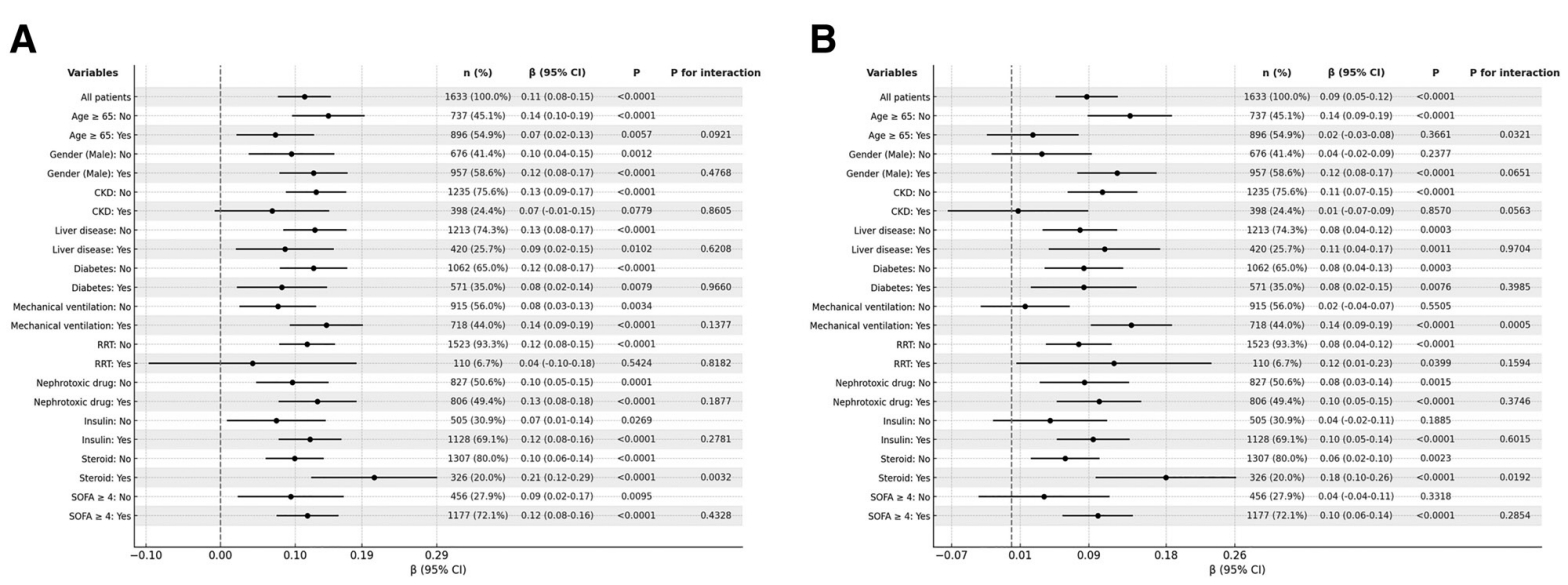
Forest‐plot subgroup analyses of the associations between log(TG_max/HDL_min) and log-transformed length-of-stay outcomes based on linear regression models (Model 3). Panels show adjusted β coefficients with 95% CI for LOS in hospital (A) and LOS in ICU (B) across prespecified subgroups. CI = confidence interval, ICU = intensive care unit.

log(TG_max/ HDL_min) was associated with prolonged hospital stay (log LOS model β = 0.11 days, 95%CI 0.08–0.15, *P* < .0001; roughly a 13% longer LOS, RLR = 1.13) as well as longer ICU stay (β = 0.09, 0.05–0.12, *P* < .0001; RLR = 1.12). This lengthening effect was observed across most subgroups with no significant interaction by diabetes or insulin (the LOS increase occurred in both diabetics and non-diabetics alike) (Figs. 6–7). However, steroid use significantly modified the effect size: patients on steroids had a much larger increase in LOS: for hospital LOS, steroid users had β = 0.21 (0.12–0.29), *P* < .0001 (RLR = 1.22), compared to β = 0.10 (0.06–0.14), *P* < .0001 (RLR = 1.12) in nonusers, with *P* < .01 for interaction. A similar amplification was seen in ICU LOS (steroid users β = 0.18 vs nonusers 0.06, interaction P ~0.02, corresponding RLR ~1.28 vs 1.08). Additionally, the ICU LOS effect was age-dependent and ventilation-dependent: the prolongation was significant in younger patients (<65 years: β = 0.14, *P* < .0001) but minimal in those ≥65 (β ~0.02, *P* = .3661; *P* interaction = .0321), and it was evident in patients who required MV (ventilated: β = 0.14, *P* < .0001) but not in those who did not (β ~0, *P* = .55; *P* interaction = .0005). In summary, log(TG_max/ HDL_min) consistently lengthened hospital/ICU stay overall, with steroid therapy notably enhancing this effect, and the ICU stay impact being more pronounced in younger, mechanically ventilated patients.

### 3.10. Sensitivity analyses

To evaluate the robustness of the associations, sensitivity analyses were conducted by sequentially excluding patients with specific comorbidities or treatments and refitting Model 3. The association between log(TG_max/HDL_min) and 1-year mortality remained statistically significant in most subgroups, including after exclusion of patients with CKD (OR 1.17, 95% CI 1.03–1.33), diabetes (OR 1.26, 95% CI 1.09–1.44), insulin use (OR 1.49, 95% CI 1.19–1.85), steroid use (OR 1.16, 95% CI 1.05–1.28), MV (OR 1.15, 95% CI 1.03–1.29), and RRT (OR 1.13, 95% CI 1.00–1.26). The association was attenuated and not statistically significant after exclusion of patients with liver disease or nephrotoxic drug exposure.

For LOS in the hospital, the RLR remained significant across all subgroups, except for a marginal attenuation when excluding insulin users. Specifically, when excluding patients with LOS in hospital <7 days, the association remained robust (Exp(β) = 1.09, 95% CI = 1.05–1.13, *P* < .0001).

For LOS in ICU, associations also remained significant across most subgroups, although the significance was slightly lost when excluding patients receiving insulin (*P* = .0547) or MV (*P* = .1672). Notably, excluding patients with LOS in ICU <7 days still yielded a significant association (Exp(β) = 1.05, 95% CI = 1.01–1.10, *P* = .0066).

In the log-transformed models, β coefficients for log(LOS in hospital + 1) and log(LOS in ICU + 1) were directionally consistent and remained significant in nearly all exclusion groups. When excluding LOS in hospital <7 days, the association for log(LOS in hospital +1) remained highly significant (β = 0.11, 95% CI = 0.08–0.15, *P* < .0001). Similarly, for log(LOS in ICU + 1), excluding LOS in ICU <7 days also showed significant results (β = 0.09, 95% CI = 0.05–0.12, *P* < .0001).

These findings indicate that the observed associations between log(TG_max/HDL_min) and adverse outcomes were generally robust across most subgroup exclusions, although the results may vary slightly based on specific clinical factors. The full results can be found in Supplementary Table 1, Supplemental Digital Content 3.

## 4. Discussion

Our study demonstrates that dysregulation of lipid metabolism, reflected by an elevated log(TG_max/HDL_min) ratio, is associated with clinical outcomes in critically ill patients with sepsis-associated AKI. This clinical observation aligns with our previous experimental findings in murine SA-AKI, where fenofibrate and ketogenic diet enhanced renal FAO, reduced lipid accumulation, and improved mitochondrial energy metabolism.^[5,6]^ Patients with higher log(TG_max/HDL_min) values showed significantly increased 1-year mortality and tended to experience longer ICU and hospital stays. This association persisted after adjusting for conventional risk factors, indicating that the log(TG_max/HDL_min) may capture additional risk by severity of illness alone. Sensitivity analyses confirmed the robustness of these associations across most clinical subgroups. The associations with hospital and ICU LOS remained consistently significant in all subgroup exclusions except for insulin use, while the association with 1-year mortality showed attenuation of significance after exclusion of patients with liver disease or nephrotoxic drug exposure, suggesting partial dependence on metabolic or treatment-related factors. Notably, the impact of an elevated log(TG_max/HDL_min) was most pronounced in the subgroup of non-diabetic, insulin-naïve patients, suggesting that baseline metabolic health modulates this risk marker’s prognostic value. In patients without preexisting insulin resistance or diabetes, acute lipid perturbations may be more discriminating for outcome, whereas in long-standing diabetics (often already dyslipidemic), the log(TG_max/HDL_min) is less dynamic and thus less predictive. These findings suggest that log(TG_max/HDL_min) may serve as a readily available biomarker associated with adverse outcomes.

Although we additionally constructed an expanded multivariable model (Model 4) that incorporated comorbidities and ICU treatment-related variables, the effect estimates for log(TG_max/HDL_min) were nearly identical to those in Model 3. Many covariates in Model 4 (e.g., vasopressor use, MV, RRT) are highly correlated with disease severity or lie on the causal pathway of SA-AKI mortality. Adjusting for such variables risks statistical overadjustment and may attenuate true associations by removing part of the biological effect mediated through critical illness progression. Furthermore, the inclusion of numerous inter-correlated covariates increased noise without improving predictive performance. Given the statistical stability of effect estimates, reduced risk of overadjustment, and superior model performance, Model 3 was selected as the primary analytical model for spline analyses, subgroup analyses, sensitivity analyses, and ROC evaluation.

The AIP has emerged as a robust prognostic marker across multiple acute and chronic conditions. In acute MI, higher AIP independently predicted increased 28-day mortality.^[16]^ Among patients with acute decompensated heart failure, those in the highest AIP quartile exhibited significantly greater 30-day mortality.^[17]^ In acute ischemic stroke, elevated AIP was associated with poorer 3-month functional outcomes.^[18]^ In severe acute pancreatitis, increased AIP conferred higher odds of critical illness and ICU admission.^[19]^ CKD cohorts demonstrated that elevated AIP predicted long-term all-cause and cardiovascular mortality.^[20]^ In hospitalized coronavirus disease 2019 patients, an AIP threshold >0.6285 accurately forecasted in-hospital death (AUC ≈ 0.85) and need for intensive care.^[21]^ Among critically ill individuals with atherosclerotic CVD, higher AIP correlated with prolonged ICU and hospital stays.^[22]^ Collectively, these recent findings underscore the AIP’s utility as a prognostic index in both ICU and broader clinical settings.

The biological plausibility of TG/HDL as a risk marker in sepsis-AKI is supported by the pathophysiological changes in lipid metabolism during critical illness. Sepsis induces a profound reprogramming of energy metabolism, shifting the body away from efficient FAO toward a more dysfunctional, lipid-storing state. Inflammatory cytokines and stress hormones promote adipose tissue lipolysis and insulin resistance, flooding the circulation with free fatty acids and TG-rich lipoproteins.^[23,24]^ The acute phase response also suppresses hepatic production of apolipoprotein A-I and inhibits lipoprotein lipase, resulting in impaired clearance of TG-rich very low-density lipoprotein (VLDL) particles and a sharp drop in HDL levels.^[24]^ As a result, septic patients often develop hypertriglyceridemia concomitant with low HDL cholesterol: an atherogenic profile that reflects both the severity of inflammation and an altered metabolic milieu. In sepsis-associated AKI, these derangements may be even more pronounced: experimental studies have shown that AKI is linked to downregulation of peroxisome proliferator-activated receptor-α (PPAR-α) and other nuclear receptors that regulate FAO.^[24]^ The loss of PPAR-α activity in sepsis impairs mitochondrial FAO, leading to accumulation of TG in tissues and further reductions in HDL (since PPAR-α also influences apoA-I expression and reverse cholesterol transport).^[24]^ This PPAR-α suppression has been implicated in the metabolic and inflammatory derangements of AKI during sepsis, and conversely, pharmacologic activation of PPAR-α has been shown to improve lipid clearance and survival in septic models.^[24]^ Beyond PPAR signaling, high TG and low HDL levels themselves can exacerbate inflammation and organ dysfunction. TG-rich lipoproteins can accumulate in organs and promote oxidative stress, while low HDL deprives the host of HDL’s anti-inflammatory and endotoxin-neutralizing functions.^[24]^ Normally, HDL particles help sequester and clear bacterial lipopolysaccharide and modulate the immune response; in their absence, the septic inflammatory cascade may be amplified.^[24]^ The excess free fatty acids and oxidative stress associated with lipid metabolic failure can damage mitochondria and impair cellular energy production in vital organs (including the kidneys), contributing to organ injury.^[24,25]^ In essence, an elevated TG/HDL ratio in sepsis-AKI can be viewed as a composite marker of this metabolic inflammation: capturing insulin resistance, impaired FAO, and HDL dysfunction. These pathophysiological insights underscore why patients with higher TG/HDL are vulnerable to worse outcomes: their metabolic response to sepsis is maladaptive, fueling inflammation and organ damage through mechanisms involving PPAR signaling, inflammatory cytokines, and mitochondrial dysfunction.

Despite its strengths, this study has several limitations. First, it was a retrospective analysis based on a single-center database (MIMIC-IV), which may limit generalizability. The analyzed cohort represented a subset of eligible SA-AKI patients with available lipid measurements, as lipid testing in the ICU is performed based on clinical indications rather than a standardized protocol. Therefore, selection bias may be present, and the findings may not be fully generalizable to all patients with SA-AKI. Second, the observational design precludes causal inference. Although we adjusted for multiple potential confounders, residual confounding from unmeasured variables: such as nutritional status (e.g., caloric intake, feeding routes, malnutrition) and incomplete data on lipid-lowering therapies: may still exist. Third, our exposure variable, log(TG_max/HDL_min), was defined using extreme lipid values within the first ICU week. While this approach was intended to capture the most pronounced metabolic disturbance during the acute phase of SA-AKI, it may be influenced by laboratory timing, measurement frequency, non-fasting conditions, and clinical interventions. Because lipid measurements were obtained as part of routine clinical care, sampling frequency was not standardized and may vary across patients. Patients with more frequent laboratory testing: often reflecting greater illness severity: are more likely to have extreme values recorded, which may artificially inflate the exposure estimate. Thus, the observed associations may partially reflect differences in monitoring intensity rather than true biological variation. Although sensitivity and subgroup analyses suggested that the observed associations were broadly consistent, this exposure definition may still overemphasize transient abnormalities. Future prospective studies with standardized lipid sampling and time-updated modeling are warranted. In addition, lipid measurements were obtained as part of routine clinical care rather than a standardized protocol, and sampling conditions (e.g., fasting status) and timing were not controlled. These factors may introduce additional variability and limit the interpretability of “extreme” lipid values. Although sensitivity and subgroup analyses suggested that the observed associations were broadly consistent, this exposure definition may still overemphasize transient or nonrepresentative abnormalities. Fourth, adjustment for peak renal indices (e.g., BUN_max and creatinine_max) may introduce potential overadjustment because these variables could partially reflect disease progression. Therefore, the estimated associations should be interpreted with caution. Fifth, although the multivariable model showed acceptable discrimination for 1-year mortality, this analysis was exploratory. No internal validation or calibration assessment was performed, and the predictive performance should therefore be interpreted cautiously and confirmed in external cohorts. In addition, although 1-year mortality status was completely available for all included patients and logistic regression was used to model fixed-time mortality risk, time-to-event approaches such as Cox proportional hazards models may provide complementary information in future studies. Finally, we focused primarily on mortality and LOS outcomes and did not evaluate longer-term end points such as post-discharge cardiovascular events or renal recovery. Prospective, multicenter studies are needed to confirm these findings and further explore the clinical implications of lipid dysregulation in SA-AKI.

## 5. Conclusions

In this large retrospective cohort, an elevated log(TG_max/HDL_min) during the first ICU week was independently associated with increased 1-year mortality and prolonged ICU and hospital stays. These findings suggest that log(TG_max/HDL_min) may serve as a readily accessible marker of extreme dyslipidemia associated with adverse outcomes in SA-AKI. Further prospective studies are needed to confirm these associations and evaluate potential clinical applications.

## Acknowledgments

We thank the Department of Critical Care Medicine at the Second Affiliated Hospital of Harbin Medical University for its support of this study.

## Author contributions

**Conceptualization:** Siyao Zeng, Hanxin Liu, Zhen Quan, Zhipeng Yao, Yu Zhang.

**Data curation:** Siyao Zeng, Hanxin Liu, Zhen Quan, Zhipeng Yao, Yu Zhang.

**Formal analysis:** Siyao Zeng, Hanxin Liu, Zhen Quan, Zhipeng Yao, Yu Zhang.

**Investigation:** Siyao Zeng, Hanxin Liu, Zhen Quan, Zhipeng Yao, Yu Zhang.

**Methodology:** Siyao Zeng, Hanxin Liu, Zhen Quan, Zhipeng Yao, Yu Zhang, Yue Li.

**Software:** Siyao Zeng, Hanxin Liu, Zhen Quan, Zhipeng Yao, Yu Zhang.

**Visualization:** Siyao Zeng.

**Project administration:** Yue Li, Junbo Zheng, Hongliang Wang.

**Validation:** Yue Li, Junbo Zheng, Hongliang Wang.

**Resources:** Junbo Zheng, Hongliang Wang.

**Writing – original draft:** Siyao Zeng.

**Writing – review & editing:** Junbo Zheng, Hongliang Wang.




















