# Supplementary figures and images for: Extreme atherogenic index of plasma (log[TG_max/HDL_min]) and prognosis in sepsis-associated acute kidney injury: A MIMIC-IV retrospective study
Source: Medicine (Baltimore). 2026 Jul 10;105(28):e49718. doi: 10.1097/MD.0000000000049718 (PMC13363196; doi:10.1097/MD.0000000000049718)

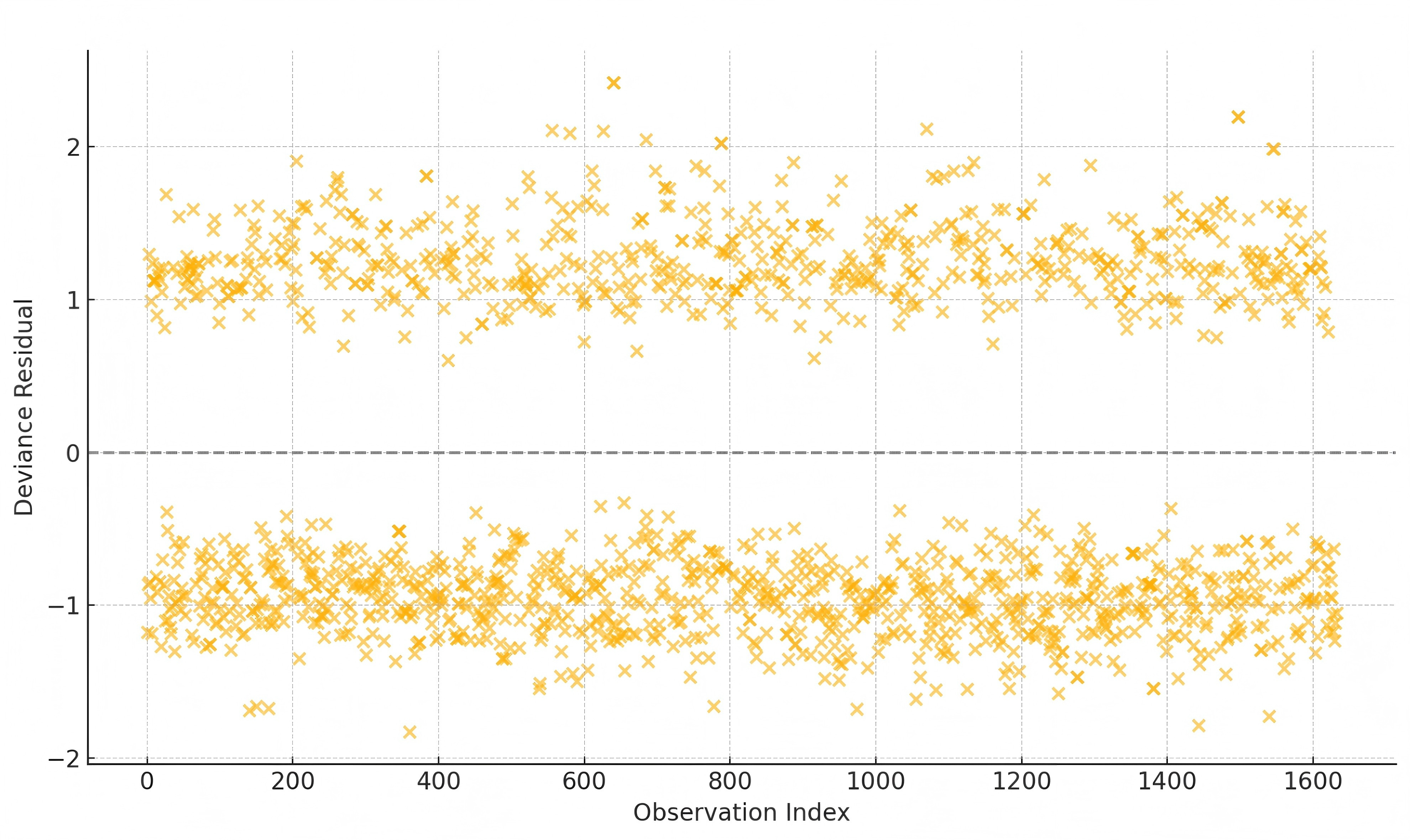

Supplement: Supplementary file 1 [file medi-105-e49718-s001.tif]

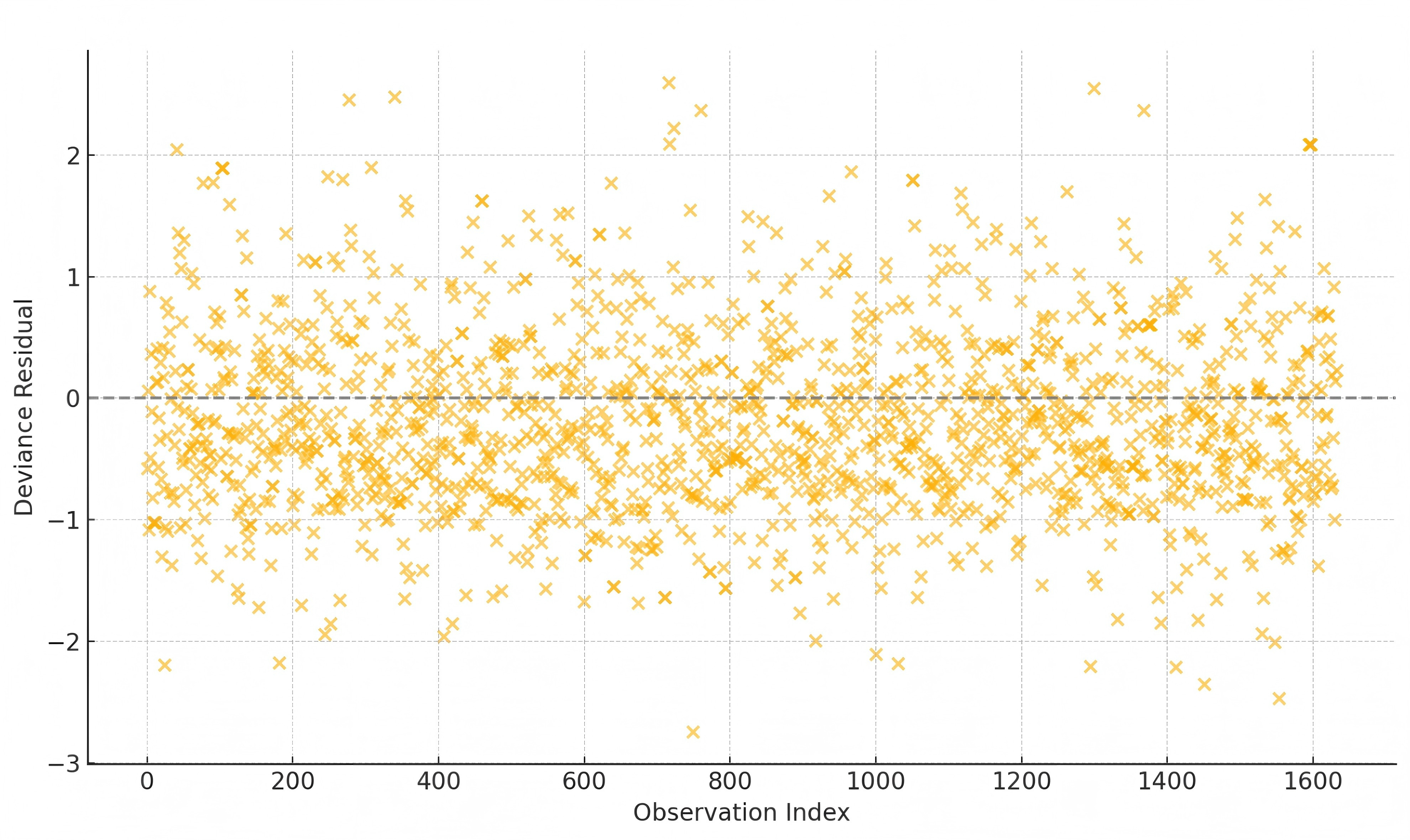

Supplement: Supplementary file 2 [file medi-105-e49718-s002.tif]

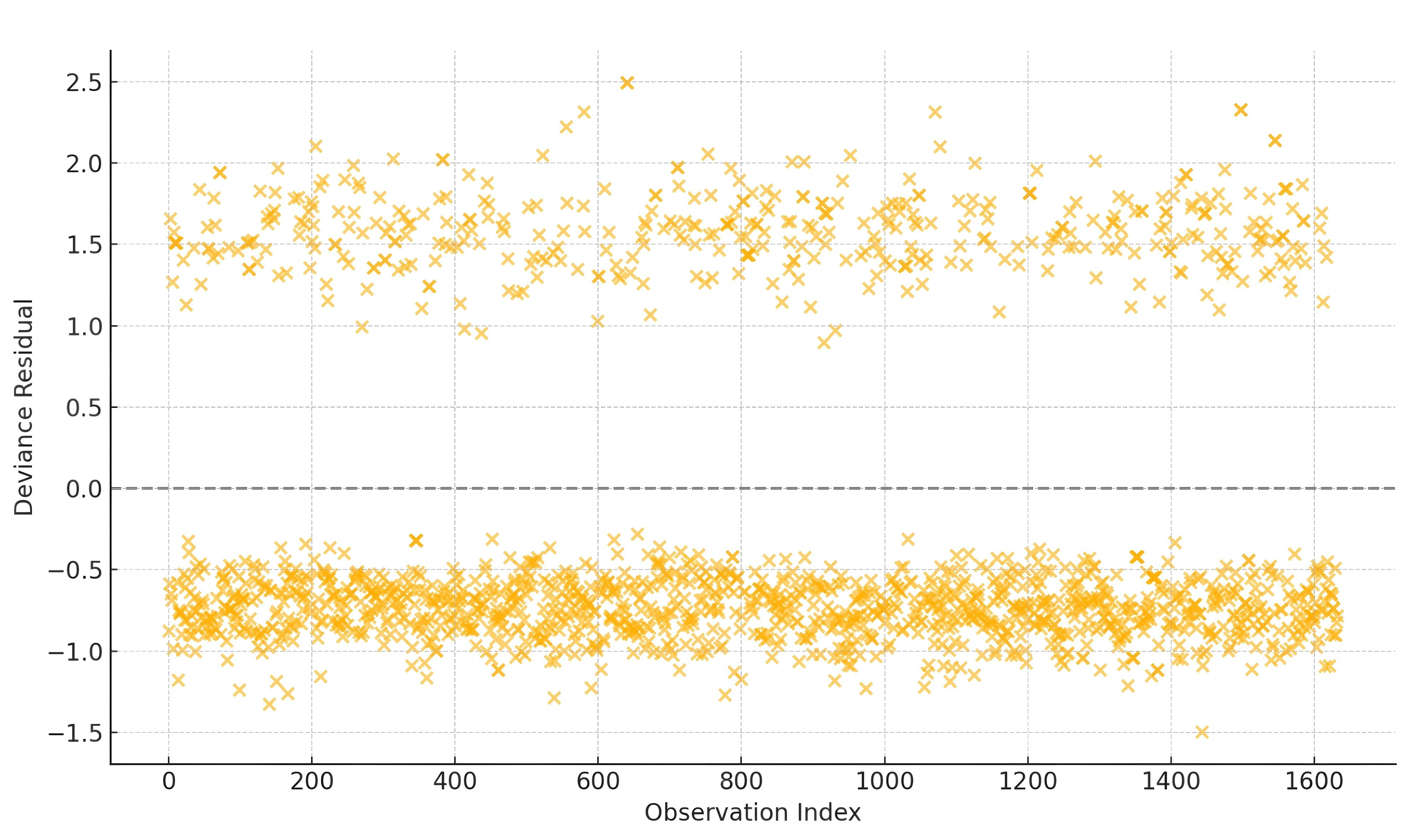

Supplement: Supplementary file 4 [file medi-105-e49718-s004.tif]

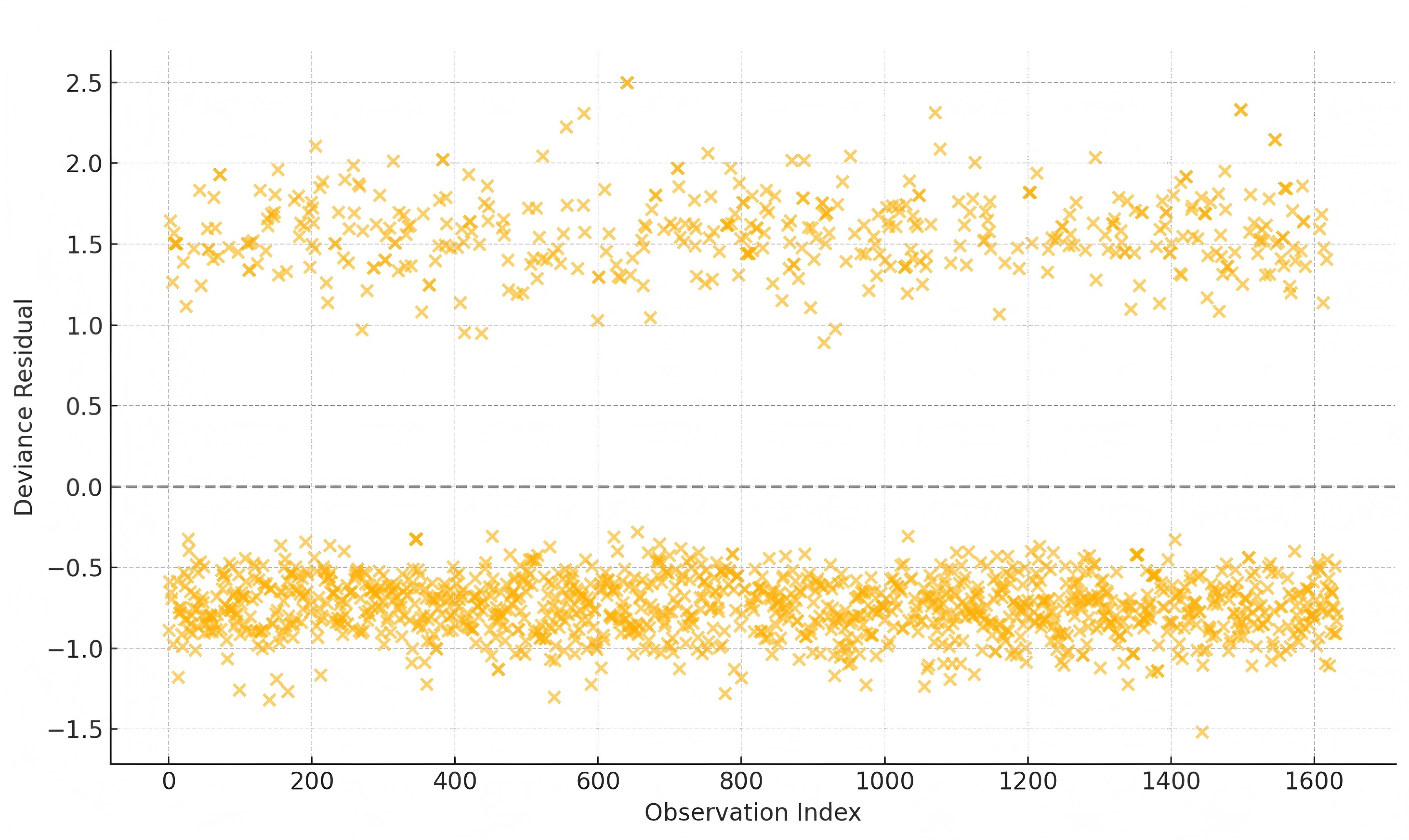

Supplement: Supplementary file 5 [file medi-105-e49718-s005.tif]

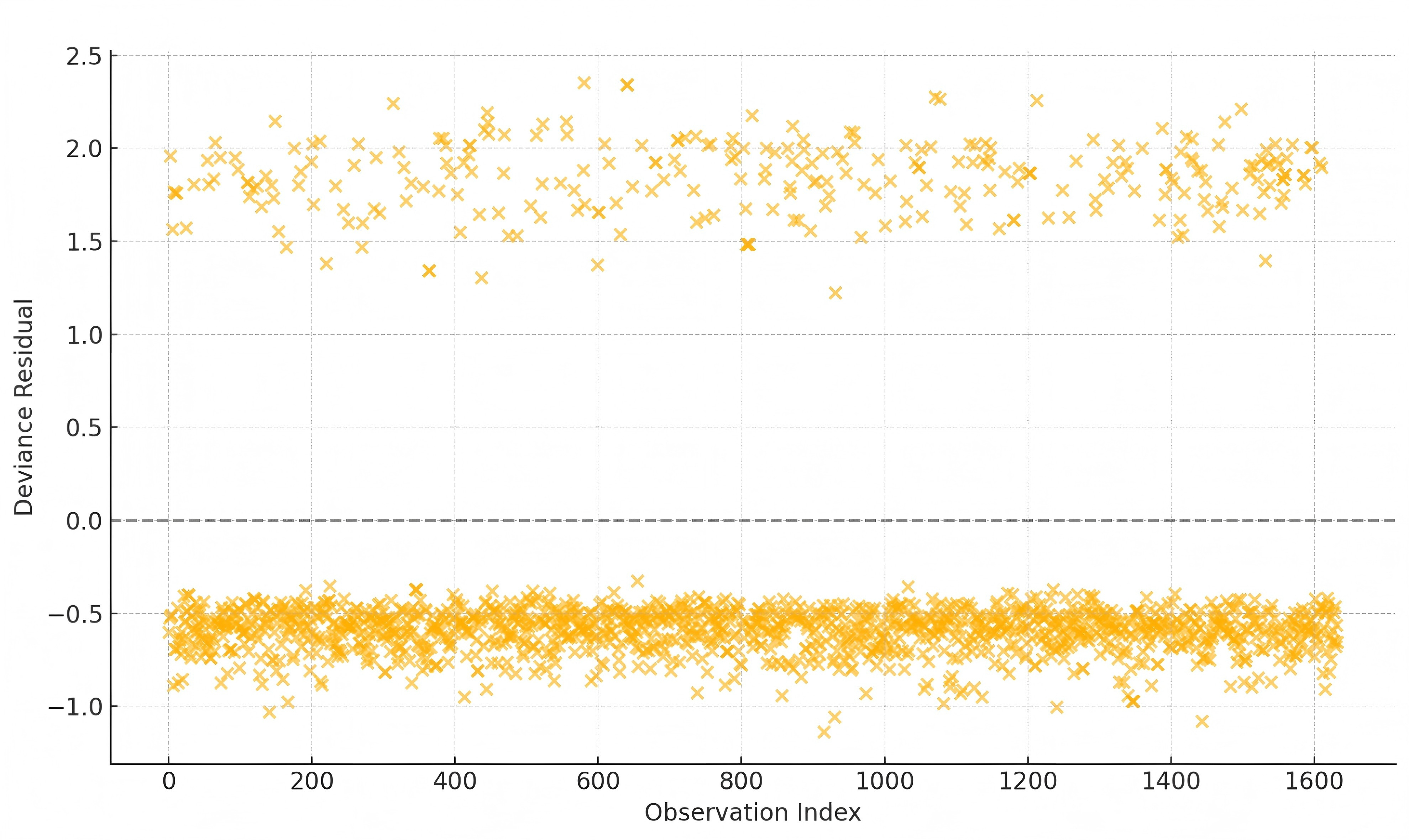

Supplement: Supplementary file 6 [file medi-105-e49718-s006.tif]

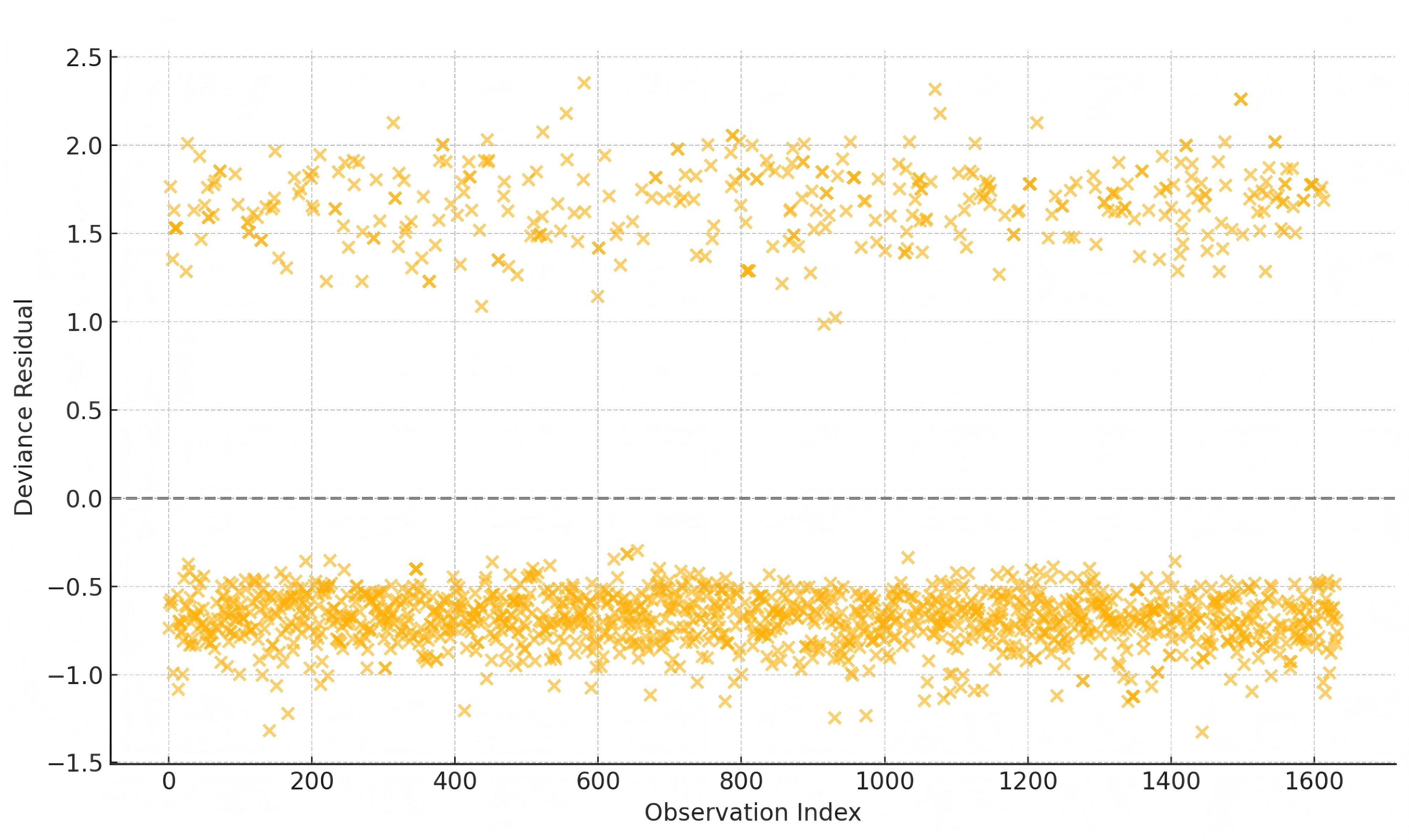

Supplement: Supplementary file 7 [file medi-105-e49718-s007.tif]

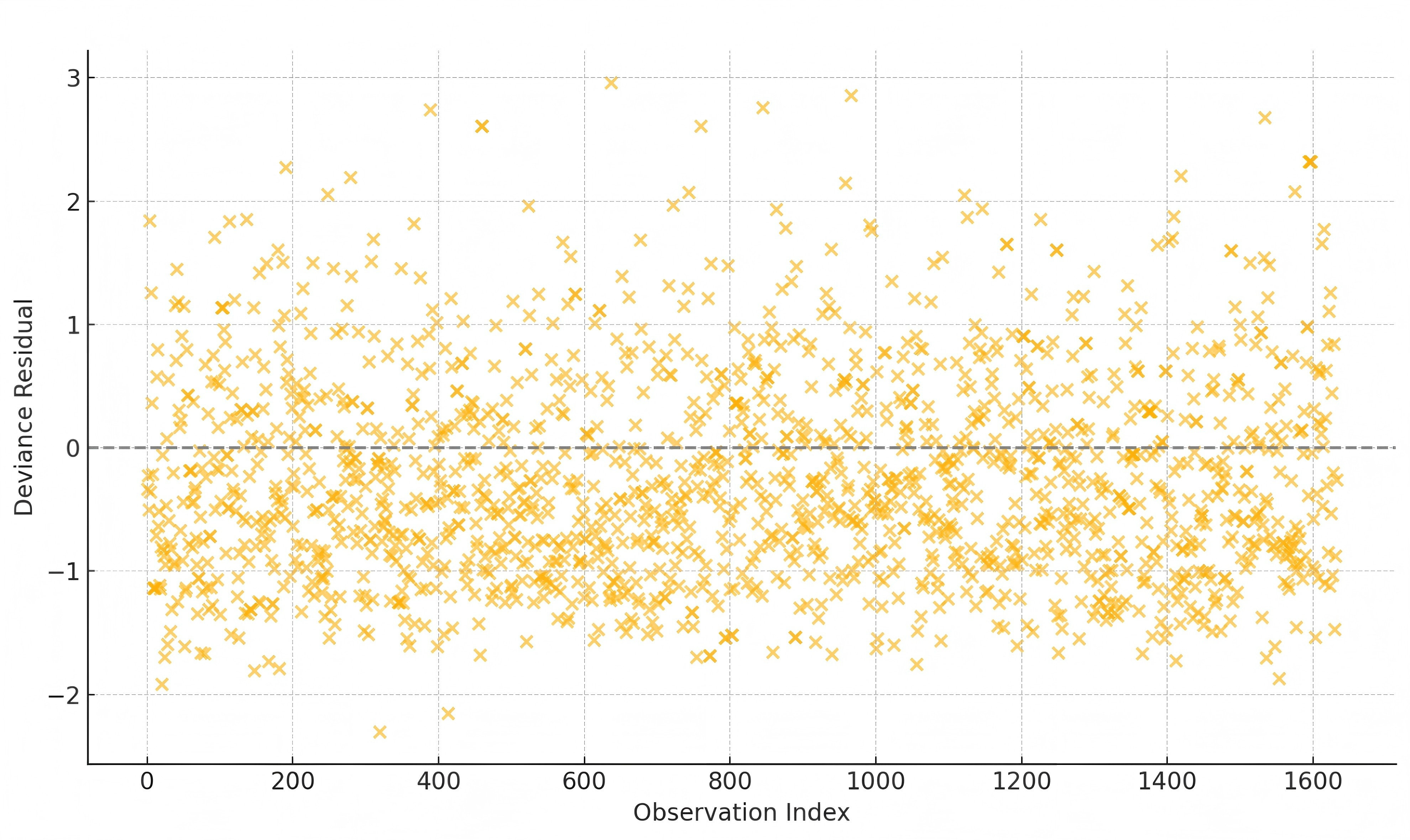

Supplement: Supplementary file 8 [file medi-105-e49718-s008.tif]

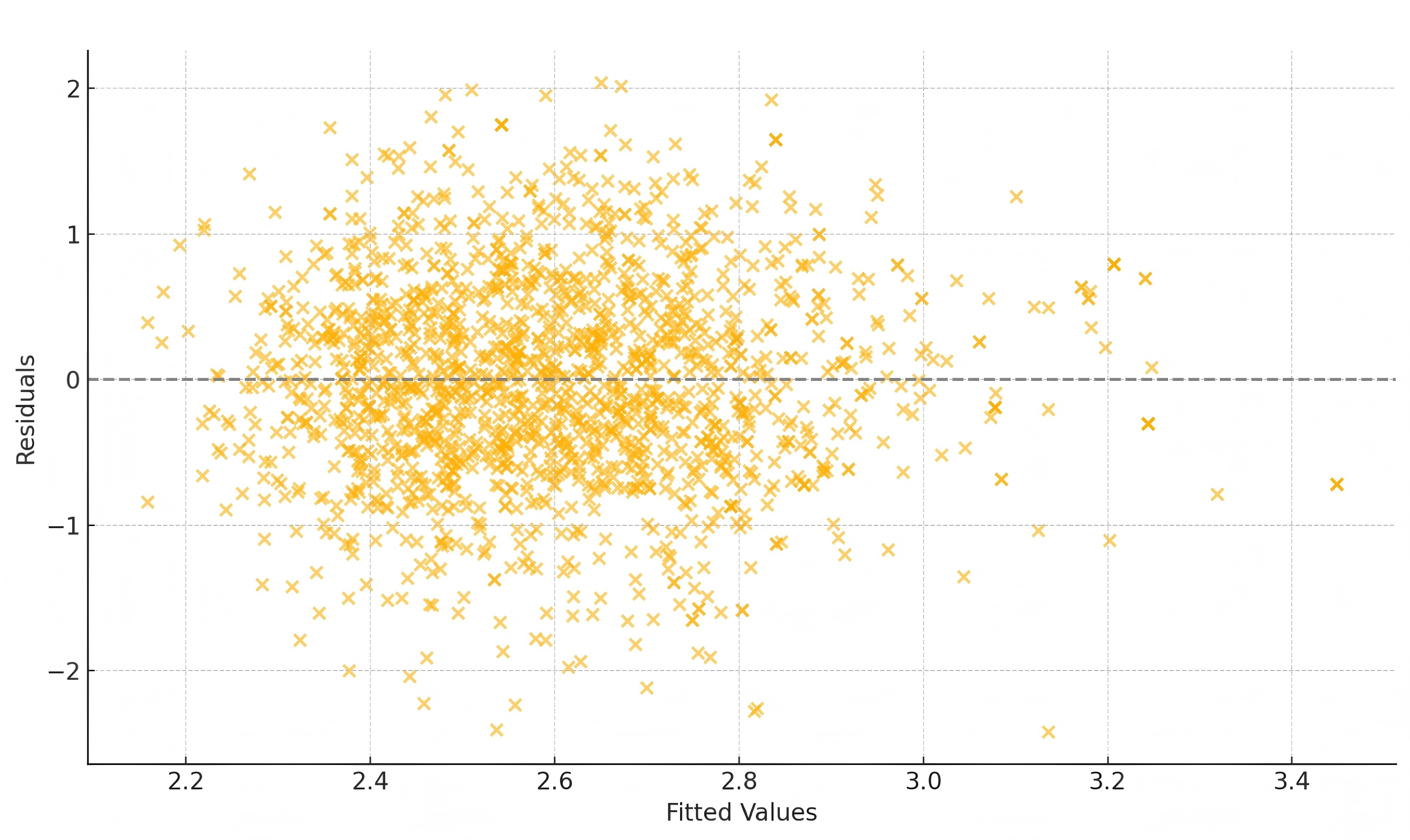

Supplement: Supplementary file 9 [file medi-105-e49718-s009.tif]

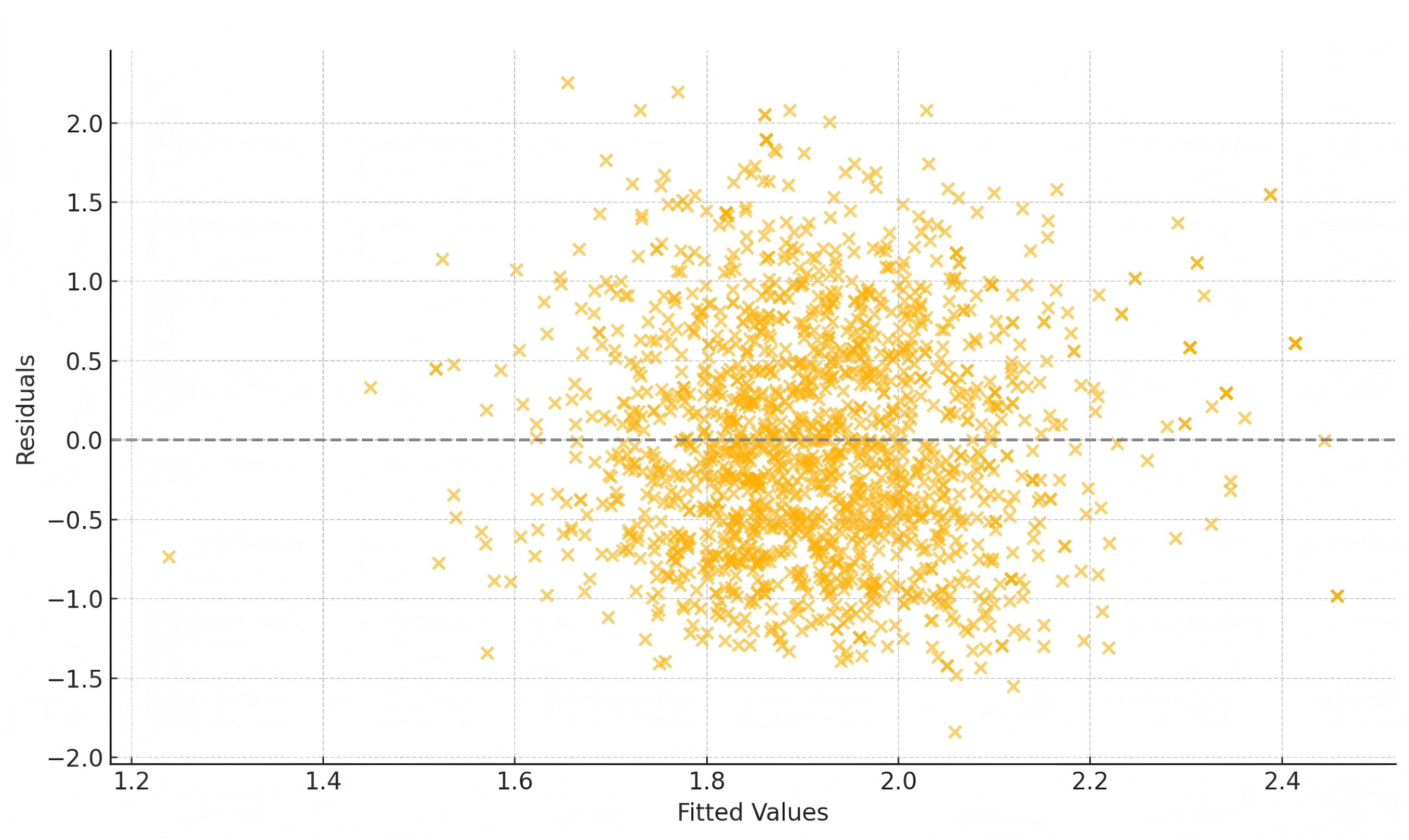

Supplement: Supplementary file 10 [file medi-105-e49718-s010.tif]
